# Epigenetic regulation of intracellular branched-chain amino acid homeostasis maintains a normal lifespan

**DOI:** 10.1016/j.isci.2025.112846

**Published:** 2025-06-07

**Authors:** Sejung Park, Yan Liu, Suji Lim, Hong-Yeoul Ryu, Seong Hoon Ahn

**Affiliations:** 1 Department of Medicinal and Life Science, College of Science and Convergence Technology, Hanyang University, Ansan, Gyeonggi-do 15588, Republic of Korea; 2 BK21 Plus KNU Creative BioResearch Group, School of Life Sciences, College of National Sciences, Kyungpook National University, Daegu 41566, Republic of Korea

**Keywords:** Properties of biomolecules, Molecular genetics, Epigenetics, Microbial metabolism

## Abstract

Cells experience a progressive decline in function and lifespan, accompanied by epigenetic changes. Here, we show that intracellular BCAA (icBCAA) homeostasis is regulated by histone H3K4 and H3K121 in budding yeast. Using a comprehensive H3/H4 mutant library, we identified residues essential for lifespan maintenance linked to BCAA metabolism. Among these, H3K4A/R and H3K121A mutations led to significant transcriptional changes in genes involved in BCAA biosynthesis and catabolism, accompanied by abnormally elevated icBCAA levels. Consistent with the upregulation of *BAT1*, *ILV6*, and *ADH1* genes in the H3K121A mutant, chromatin immunoprecipitation revealed increased H3K4me3 at their promoters. The genetic perturbation of *BAT1* and *BAT2* restored icBCAA balance and partially rescued lifespan defects in H3K4 or H3K121 mutants. Additionally, H3K4 and H3K121 mutations affected lifespan regulation through TORC1 signaling. Our findings suggest that the epigenetic control of BCAA metabolism, specifically through the modification of histone residues, contributes to maintaining metabolic homeostasis and replicative lifespan.

## Highlights

• Lifespan is affected by mutations of histone H3/H4 across geographical domains
• BCAA metabolism regulation rescues lifespan defects in H3K4 or H3K121 mutants
• Lifespan regulation through H3K4 or H3K121 modulation involves TORC1 signaling
• H3K4 and H3K121 are essential for intracellular BCAA homeostasis and normal lifespan

## Graphical abstract

**Figure:**
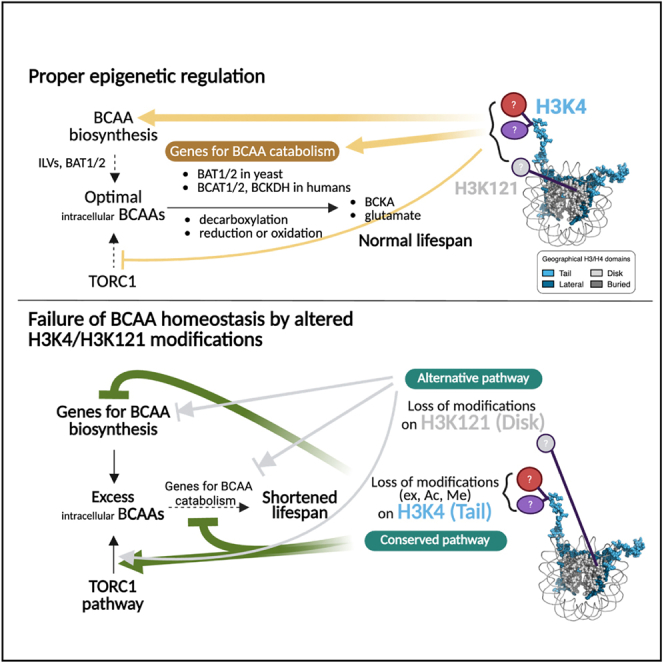

## Introduction

Aging is a time-dependent functional decline affecting most living organisms.^1^ Recent advances in aging research suggest that aging is not solely the result of genomic instability over time—mainly linked to double-stranded DNA breaks—but also by the progressive accumulation of cellular vulnerabilities, including changes in epigenetic information. Many studies support the idea that losing epigenetic information is the leading cause of aging; members of the sirtuin family of NAD-dependent histone deacetylase have been studied extensively as potential anti-aging factors, and the overexpression of Sir2 and sir-2.1 extends the replicative lifespan (RLS) in *Saccharomyces cerevisiae* and *C. elegans*, respectively.^2^^,^^3^^,^^4^ In mammals, at least three members of the sirtuin family, *SIRT1*, *SIRT3*, and *SIRT6*, are regarded as pro-longevity factors, contributing to healthy aging.^5^^,^^6^^,^^7^ Moreover, the overexpression of histones or deletion of *SET2*, a histone H3K36 methyltransferase, extends yeast lifespan, indicating that epigenetic information regulates lifespan.^8^^,^^9^ This view of aging aligns with the hypothesis that eukaryotic cells progressively lose transcriptional networks and epigenetic information as they age.^10^^,^^11^

The BCAAs include leucine (Leu), isoleucine (Ile), and valine (Val). These are essential amino acids in humans and play important roles in nutrient sensing and cellular signaling.^12^ In addition, BCAAs are not merely protein components but are also implicated in regulating lifespan, metabolic health, and tumorigenesis; protein-restricted and BCAA-restricted diets improve metabolic health in mice.^13^ In mice, the long-term consumption of high-BCAA diets induces hyperphagia, obesity, and reduced lifespan by disrupting the balance between BCAAs and other amino acids, notably tryptophan and threonine.^14^ Similarly, reduced Ile or Val promotes metabolic health in mice, and dietary levels of Ile are positively associated with BMI in humans.^15^ Furthermore, a multi-omic approach revealed that BCAA catabolism is lost during tumor development and progression while simultaneously exhibiting increased intracellular BCAA accumulation.^16^ Accumulating evidence further suggests the theory that the persistent activation of mammalian target of rapamycin complex 1 (mTORC1) links increased plasma BCAA levels to insulin resistance. Excess nutrient-driven obesity elevates plasma levels of both Leu and insulin, together with the activation of ribosomal protein S6 kinase 1, S6K1.^12^^,^^17^^,^^18^

In the present study, we explored the role of the epigenetic histone marks H3K4 and H3K121 in regulating cellular lifespan. We found that mutations of histone H3/H4 residues profoundly affect lifespan across geographical domains. In addition, we showed that optimal icBCAA levels are critical for maintaining a normal lifespan; modulating BCAA metabolism in cells bearing mutations on H3K4 or H3K121 partially restored their lifespan defect. Our study provides insights into the development of therapeutics for human diseases linked to BCAA metabolism and histone H3K4 or H3K121 modifications.

## Results

### Long- or short-lived histones H3 or H4 strains were identified using a modified iterative strategy for the replicative lifespan analysis

**Figure 1:**
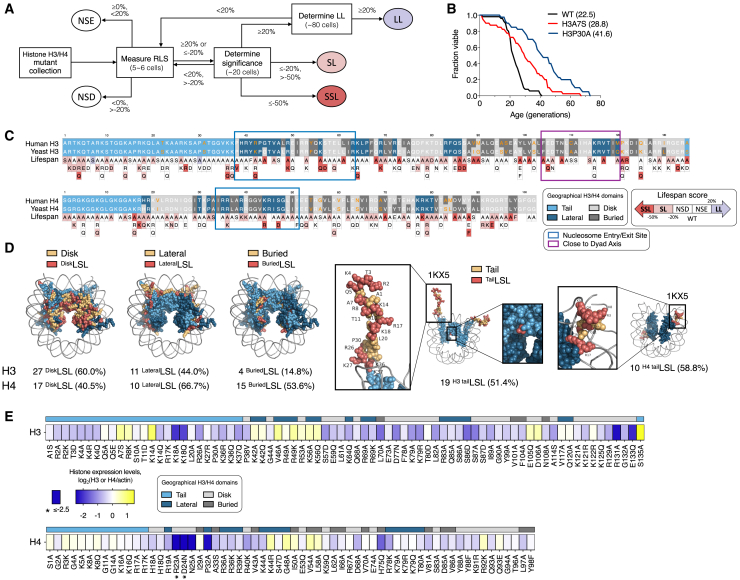
An iterative strategy for the replicative lifespan screen identified long- or short-lived cells that convey mutations in various geological domains in histone H3 or H4 (A) Schematic representation of the RLS screening strategy used in this study. The indicated mutant cells from the yeast synthetic histone H3 and H4 mutant collection (Open Biosystems) were categorized into long-lived (LL) (≥+20% increase in RLS), short-lived (SL) (≤−20%, > −50% decrease in RLS), and significantly short-lived (SSL) (≤−50% decrease in RLS) based on their mean lifespan relative to the wild-type. The percentage values represent the percentage change in lifespan. NSE refers to “no significant extension,” while NSD refers to “no significant decrease.” (B) RLS analysis for H3A7S and H3P30A strains, which are determined as “LL” strains. More than fifty cells were used for the experiment analyzed to determine mean lifespan. Each mean lifespan is shown in parentheses. Statistical significance was determined using the Mann-Whitney test (∗*p* < 0.05, ∗∗*p* < 0.01, ∗∗∗*p* < 0.001). (C) Geographical distribution of the substitution residues in the long- or short-lived (LSL) cells. Residues that convey mutations in various geographical domains in histone H3 or H4 were highlighted in indicated colors. (D) (D) The histone H3/H4 tetramers from PDB: 1KX5 (*Homo sapiens*) and PDB: 4JJN (*Saccharomyces cerevisiae*) were shown with highlighted geographical domains and the LSL residues within them. The same color codes as in Figure 1C were used. Nucleosome structures were modeled using PyMOL (Version 2.0, Schrödinger, LLC) with the PDB entries 4JJN and 1KX5 as reference structures. (E) Effect of LSL strains on the expression levels of histone H3 or H4. Histone levels were assessed in wild-type and mutant strains by immunoblotting, with actin used as a loading control. Expression levels are color-coded from dark blue (low) to bright yellow (high) and mapped to their geographical distribution.

To determine which amino acid residues in the histone H3 or H4 are associated with regulating cellular lifespan, we performed the RLS analysis with the yeast non-essential histone H3 and H4 mutant collection using a modified iterative strategy of the RLS screen (Figure 1A).^19^ The properties of the substitution of each amino acid residue are as described previously.^20^ We modified the previous strategy to improve the accuracy and confer significance to the short- or long-lived strains (referred to as SL or LL; see STAR Methods for details) by increasing the mother cells tested. We also refer to the histone H3/H4 mutant strains, which shorten or extend lifespan, as the long- or short-lived (LSL) strains hereafter. The RLS screen revealed that more than 40% of the LSL substitutions of the histone H3 or H4 are associated with lifespan regulation (Figure S1A). We found 2 LL, 93 SL, and 43 significant SL (SSL) strains from H3/H4 substitution mutants (Figure S1A). Among them, two LL strains, either H3A7S or H3P30A cells with a mutation on the 7th alanine (Ala) or the 30th proline (Pro) residues of histone H3, confirmed their ability to extend lifespan with an increased number of cells up to 80 (Figure 1B). Intriguingly, unlike the P38A/P39V mutations on histone H3, which affect the trimethylation of K36 on histone H3,^21^ mutations of H3A7S or H3P30A had little effect on H3 methylation at K4 or K36 (Figure S1B).

### A geographical analysis of the LSL strains shows that lifespan is affected by mutations of histone residues across the H3/H4 tetrasome

Previously, it was reported that either the nucleosome entry/exit sites^22^ or the residues close to the dyad axis, in which four of the histone dimers form an octamer with a 2-fold symmetry axis,^23^^,^^24^ were the residues affected by lethal substitution mutations to the nucleosome structure.^20^ However, our screen results display the unbiased distribution of the LSL residues across the histone H3/H4 sequences (see Figure 1C). This prompted us to confirm whether each location of the LSL residues is not confined to the distinct structure of a nucleosome. To delimit nucleosomal regions, we assigned each histone H3/H4 residue similarly to previously reported.^20^ Consistent with the equitable distribution of each LSL residue across histone H3/H4 sequences, we found that the LSL residues constituted more than 40% of the disk, lateral, or tail domains of the (H3/H4)_2_ tetramer (Figure 1D). In addition, as many as 4 or 15 residues within the buried domain of (H3/H4)_2_ tetramer include the LSL phenotype. Thus, these results show that the lifespan-associated residues of (H3/H4)_2_ tetramer are broadly distributed throughout geographical domains of the histone H3 or H4.

Next, we investigated whether substitution mutations in different nucleosomal domains affect histone stability. Histone levels have previously been reported to decrease with cellular aging.^8^ We also found that mutations in LSL strains that neutralize either positive (H3K18A, H3R131A, and H4R23A) or negative charges (H3E133Q and H4D24N) significantly reduced protein levels (Figures 1E, S2A, and S2B).

Notably, except for H3K18A, located on the tail domain, most acid-to-neutral or basic-to-neutral strains belong to the disk domain. Interestingly, charge-neutralizing mutations in residue pairs—H3R131A/H3E133Q at the C-terminus of H3 and H4R23A/H4D24N at the N-terminus of H4—led to a marked reduction in protein levels, approaching background levels. Thus, the results show that the integrity of histone residues, mainly located on the disk domain, is critical for maintaining a normal lifespan.

### Many LSL strains have growth sensitivity to sulfometuron methyl

**Figure 2:**
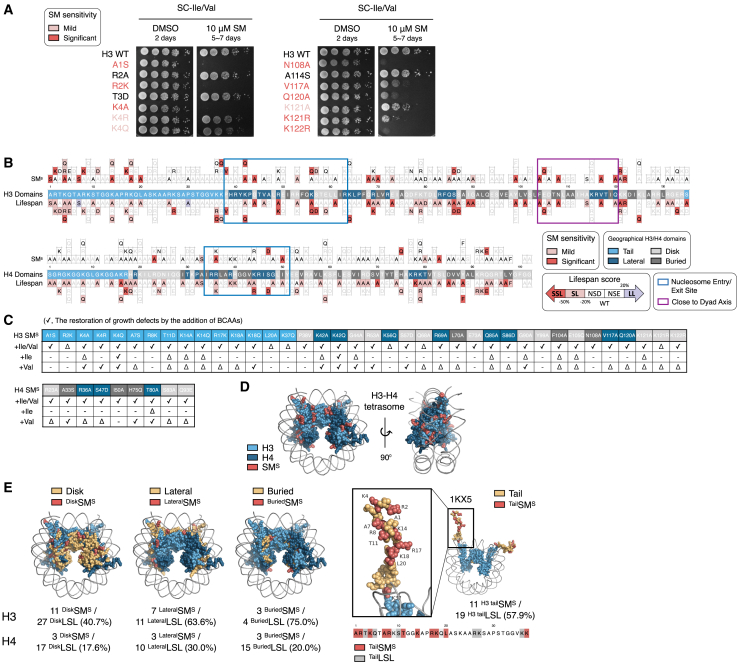
Starvation of Ile and Val by the treatment of SM identified that SM-sensitive LSL cells with severe defects in their growth are distributed across geographical domains (A) Cells with WT background or the indicated LSL mutations were incubated with vehicle DMSO or 10 μM of SM for the indicated days. Colors represent growth sensitivity: red refers to significant SM sensitivity, while light red refers to mild sensitivity. (B) Mapping of SM-sensitive LSL residues (top), geographical domains of histone H3/H4 (middle), and their corresponding lifespan scores (bottom) is shown. (C) Growth restoration of SM-sensitive mutants by Ile and Val. Cells were supplemented with Ile (0.8 mM) or Val (0.89 mM) to determine their ability to rescue SM-sensitive growth defects; checks represent full recovery, triangles indicate partial recovery, and crosses denote no recovery. (D and E) Geographical mapping of SM-sensitive LSL residues on the H3-H4 tetramer. Red dots indicate SM-sensitive residues, and the geographical domains in histone H3 or H4 were highlighted as in Figure 1C.

We sought to explore the possible mechanisms through which the lifespan of LSL strains was influenced. To this end, we treated various drugs that inhibit many steps in regulating gene expression and explored whether they cause growth defects in LSL strains. Contrary to our expectation, the growth of many LSL cells was highly sensitive solely to sulfometuron methyl (SM) (Figures 2A and S3). SM inhibits Ilv2, which causes starvation for Ile and Val in *S. cerevisiae.*^25^ We identified 45 histone H3/H4 mutants with mild or significant sensitivity against SM (Figure 2B).

We next examined whether the growth defects of SM-treated LSL strains were due to reduced intracellular levels of Ile or Val. As expected, most LSL strains grew as wildtype on SC media containing Ile and Val, even in the presence of SM (Figures 2C and S4). Noticeably, we found that the growth defect of many SM-sensitive LSL strains was fully or partially restored by supplementing Val rather than by Ile. The biosynthesis of Ile or Val involves multiple sequential enzymatic steps (see Figure 4A), and each LSL strain may affect distinct steps depending on the role of each histone residue in the expression of distinct enzymes in BCAA biosynthesis. Interestingly, while excess supplementation of Ile or Val generally caused severe growth defects of LSL strains regardless of SM treatment, several LSL cells, such as the H3K4A, H3K4R, H3K18Q, H3K56Q, H3S86D, H3Q120A, or H4R36A, were resistant to even high doses of Ile or Val (Figure S4). The SM-sensitive H3 LSL cells were much more than H4; more than half of the H3 LSL cells in each domain were sensitive to SM, except those with mutations in the disk domain (Figures 2D and 2E).

### Mutations on histone H3 at K4 or K121 unusually elevated intracellular levels of BCAAs, paralleled by their shortened lifespan

**Figure 3:**
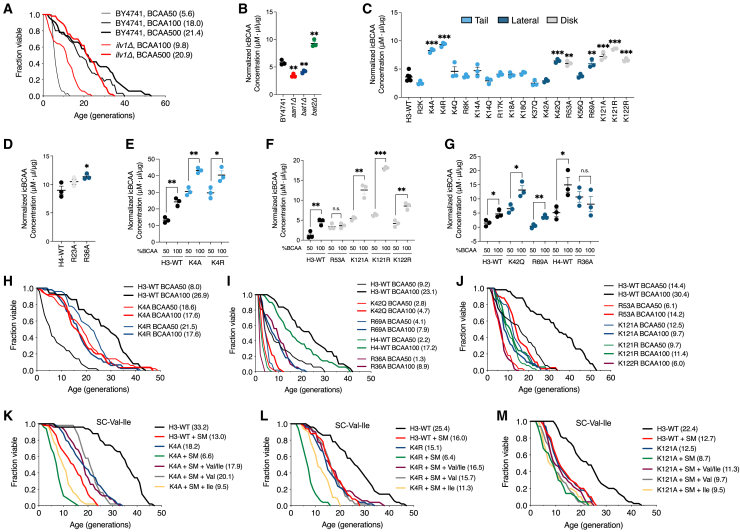
Suppression of abnormally high BCAA levels in K4 or K121 mutants of histone H3 is associated with regulating their normal lifespan (A) RLS analysis of WT and *ilv1Δ* strains supplemented with various BCAA concentrations. BCAA100 indicates the 100% BCAA concentrations of SC media: Ile (0.8 mM), Val (0.89 mM), and Leu (0.89 mM). BCAA50 or BCAA500 indicates 50% or 500% of the normal BCAA concentrations of SC media. Statistical significance was determined using the Mann-Whitney test (∗*p* < 0.05, ∗∗*p* < 0.01, ∗∗∗*p* < 0.001). (B) Quantification of free icBCAA concentrations of *aan1Δ*, *bat1Δ*, and *bat2Δ* strains. Statistical significance was determined using the t-test (∗*p* < 0.05, ∗∗*p* < 0.01, ∗∗∗*p* < 0.001). (C and D) Quantification of free icBCAA concentrations of the SM-sensitive LSL cells mutated on Arg or Lys residues within histone H3 (C) or H4 (D). (E–G) Effect of the reduction of the BCAA supplementation on icBCAA levels of the indicated SM-sensitive LSL cells. Error bars: SEM for panels (B)–(G). (H–J) Effect of the reduction of the BCAA supplementation on RLS in the indicated SM-sensitive LSL cells. (K-M) RLS analysis of WT, K4A, K4R, and K121A strains supplemented with indicated BCAA with or without 10 μM of SM treatment.

We were then prompted to determine if the levels of icBCAAs can directly modulate cellular lifespan. When wild-type (WT) yeast cells were grown in synthetic complete (SC) media containing 50% of the standard BCAA concentration (BCAA50), their RLS was markedly reduced. A similar reduction was observed in *ilv1Δ* cells lacking *ILV1*, a gene involved in Ile biosynthesis.^26^ In contrast, the complete replenishment of BCAA levels fully rescued the shortened lifespan of *ilv1Δ* cells, whereas supplementing WT cells with 500% BCAA had no significant effect (Figure 3A). The apparent lifespan extension in BY4741 upon excess BCAA or Leu supplementation (Figures 3A and S5A) is attributable to the deletion of *LEU2* in its genetic background, since BY4709, which retains functional *LEU2*, exhibited no difference in lifespan when grown with excess Leu (Figure S5B). Based on the initial observations, we found that levels of icBCAAs are critical for maintaining a normal lifespan.

Recently, a genome-wide screen and transcriptome analysis revealed that a yeast gene *AAN1* (actin, aging, and nutrient modulator protein 1) modulates the actin cytoskeleton and yeast lifespan; the deletion of *AAN1* increased actin cable stability and lifespan and was associated with decreased BCAA levels.^27^ Consistent with the report, we found that the deletion of either *AAN1* or *BAT1*, a gene that is preferentially involved in BCAA biosynthesis, lowered icBCAA levels, while loss of *BAT2*, which is preferentially involved in BCAA catabolism,^28^ conversely elevated lifespan (Figure 3B).

We next examined whether LSL strains affect icBCAA levels. Histones H3 and H4 are rich in positively charged residues such as arginine (Arg) and lysine (Lys), which are widely distributed across the nucleosomal domains of LSL and SM-sensitive LSL strains (Figure S6A). Notably, SM-sensitive LSL mutations in histone H3 were significantly enriched in Arg and Lys residues, comprising 37.7% and 40.6% of the mutated positions, respectively (Figures S6A and S6B), suggesting that the growth sensitivity to BCAA inhibition might be related to Arg/Lys dysregulation. We therefore explored whether these mutations influence icBCAA levels. Indeed, 9 out of 21 LSL strains with Arg or Lys substitutions in histone H3 or H4 showed significantly elevated icBCAA levels (Figures 3C and 3D). Moreover, the elevated icBCAA levels in these strains were normalized by reducing BCAA supplementation to 50%, except for H3R53A and H4R36A (Figures 3E–3G). Consistent with these findings, the reduced lifespan caused by mutations at H3K4 or H3K121 was not further decreased when icBCAA levels were lowered to 50% (Figures 3H–3J).

Finally, consistent with the BCAA add-back results in Figure 2C, SM treatment reduced the lifespan of H3K4A, H3K4R, and H3K121A strains. However, this effect was fully rescued by Val and Ile supplementation, with Val being more effective (Figures 3K–3M). These results strongly suggest that epigenetic regulation—particularly at H3K4 and H3K121—plays a critical role in modulating icBCAA levels to maintain normal lifespan.

### Genes related to BCAA metabolism are modulated by H3K4 or H3K121 mutations

**Figure 4:**
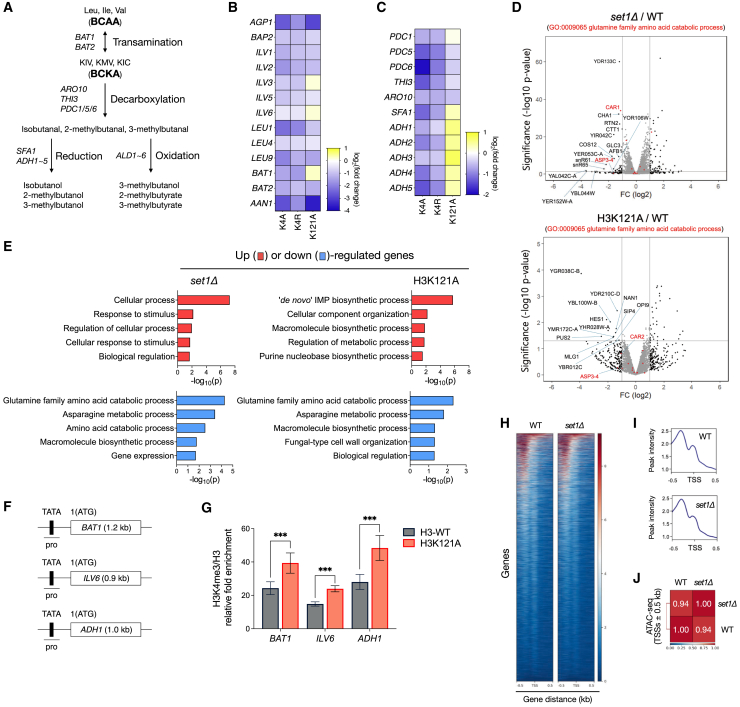
Impairment at H3K4 or H3K121 residues affects the expression of genes in BCAA metabolism (A) Schematic diagram of BCAA metabolism in yeast. In *S. cerevisiae*, BCAA metabolism is initiated by Bat1 and/or Bat2 by converting BCAAs into BCKAs. Meanwhile, Bat1 and/or Bat2 can also convert BCKAs into BCAAs. KIV, α-keto-isovalerate; KMV, α-keto-β-methylvaleric; KIC, α-keto-isocaproate. (B and C) Heatmap visualization of the RT-qPCR analysis of mRNA levels in H3K4A, H3K4R, or H3K121A cells. The relative mRNA abundance is the color-coded heatmap for BCAA biosynthetic (B) and catabolic (C) genes. Gene expression values from two biologically independent replicates are color-coded, ranging from dark blue (low expression levels) to bright yellow (high expression levels). Results were normalized to the *ACT1* level. The oligo sequences used for RT-qPCR are shown in STAR Methods. (D) Volcano plots (D) and GO enrichment analysis (E) of the genes in *set1Δ* and K121A strains were obtained from analysis using previous RNA-seq experiments. The y axis is the mean of the negative logarithm of the *p*-values, and the x axis corresponds to the log2 fold change value. Gray horizontal and vertical lines indicate a log2 fold change of −1 and 1, respectively. Red dots represent genes enriched in GO terms related to the glutamine family amino acid catabolic process. (E) Lists of differentially expressed genes were acquired by filtering the data |fold change| >1. Bar diagrams indicate the fold-enrichment of the top 5 GO biological process terms in the up (red)- or down (blue)- regulated genes. (F) Schematic diagram of *BAT1*, *ILV6*, and *ADH1* genes. The TATA box/promoter (pro) and ORF are represented by black and white boxes, respectively. Bars below the genes indicate the relative positions of the PCR products used in the ChIP-qPCR analysis. (G) H3K4me3 ChIP analysis in H3K121A strain. H3K4me3 level was normalized to H3 values and INPUT. Error bars represent the mean value ±SD of triplicate experiments from two independent chromatin preparations. Asterisks indicate statistically significant differences between the corresponding samples using a two-tailed Student’s t-test (∗*p* < 0.05; ∗∗*p* < 0.01; ∗∗∗*p* < 0.001). (H and I) The heatmap (H) and profile plot (I) of ATAC-seq signals on TSSs ± 0.5 kb regions. (J) Spearman’s rank correlation coefficient of ATAC-seq signals according to (H), the coefficient values for pairwise comparisons between WT and *set1Δ* were 0.94.

Next, we wondered if the gene expression associated with maintaining the optimal levels of icBCAA was disrupted by mutations on K4 or K121 of histone H3. BCAAs are synthesized from pyruvate (Leu and Val) or threonine (Ile). This BCAA synthesis requires a series of enzymatic activities by *ILVs* followed by *BAT1/2* transamination. Several *LEU* genes are additionally necessary for Leu synthesis.^29^ By contrast, the catabolic process of icBCAAs, known as the Ehrlich pathway, includes *BAT1/2* transamination and decarboxylation followed by reduction or oxidation (Figure 4A).^30^

The targeted RT-qPCR experiments for the genes involved in the synthesis or catabolism of BCAAs identified that most expression levels of the genes associated with optimal levels of icBCAA were downregulated by H3K4A/H3K4R mutations. However, we also observed that H3K121A disrupted genes for BCAA synthesis or catabolism in a gene-specific manner; for example, either *ILV1/2/5* was unregulated, whereas *ILV3/6* was upregulated. Similarly, *PDC5/6* for BCAA decarboxylation was downregulated, but all the *ADHs* for BCAA reduction were upregulated (Figures 4B and 4C).

Among genes involved in constant icBCAA synthesis or metabolism, *BAT1/2* are commonly involved in both pathways; Bat1 is the mitochondrial BCAA aminotransferase that is preferentially involved in BCAA biosynthesis, whereas Bat2 is a cytosolic BCAA aminotransferase preferentially involved in BCAA catabolism.^30^ As such, we examined if the overexpression of *BAT2* or the deletion of *BAT1* could lower abnormally elevated icBCAA levels observed in H3K4A/R and H3K121A cells.

To further understand whether mutations at the K4 or K121 residue of histone H3 affect the expression of genes involved in BCAA synthesis or metabolism, we re-analyzed RNA-seq data from *set1Δ* and H3K121A strains, as previously reported.^31^^,^^32^ The results revealed that the expression of *CAR1* and *ASP3-4*—classified under the glutamine family amino acid catabolic process—was significantly altered in *set1Δ* or H3K121A mutant strains. *SET1* encodes the methyltransferase responsible for H3K4 methylation (Figure 4D). In parallel, GO enrichment analysis highlighted the downregulation of glutamine (Gln) and asparagine (Asn) catabolic pathways in *set1Δ* and H3K121A mutant strains (Figure 4E).

We next determined the enrichment of H3K4 trimethylation (H3K4me3), an epigenetic mark of active transcription, at the promoters of *BAT1*, *ILV6*, and *ADH1* (Figure 4F). Consistent with results in Figures 4B and 4C, chromatin immunoprecipitation showed that H3K4me3 levels were significantly elevated at all three loci in H3K121A cells (Figures 4F and 4G).

We further sought to determine the genome-wide chromatin accessibilities in WT and *set1Δ* cells. To profile open chromatin structures in *set1Δ*, we re-analyzed ATAC-seq data.^33^ However, the heatmaps and peak intensity profiles revealed nearly indistinguishable chromatin accessibility patterns at transcription start sites (TSSs) between WT and *set1Δ* (Figures 4H–4J). Although the RNA-seq re-analysis did not directly highlight genes involved in BCAA synthesis or catabolism, our targeted RT-qPCR or ChIP assays demonstrated that key genes associated with BCAA metabolism were significantly modulated in H3K4A, H3K4R, and H3K121A strains.

### Modulation of branched-chain amino acids metabolism partially restores defective lifespan caused by H3K4 or H3K121 mutations

**Figure 5:**
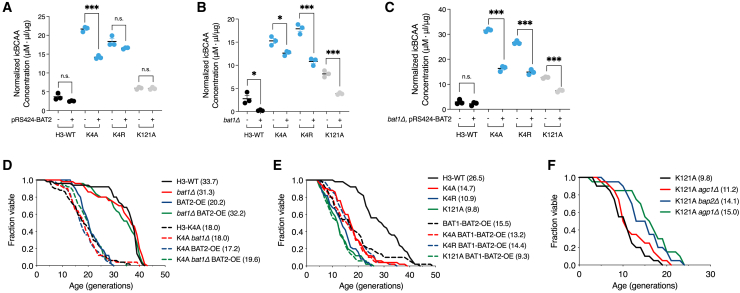
BCAA metabolism is closely associated with epigenetic lifespan regulation through histones H3K4 or H3K121 (A–C) Quantification of free intracellular BCAA levels in WT, H3K4A, H3K4R, and H3K121A strains under three conditions: (A) with or without transformation with pRS424-BAT2, (B) with or without the deletion of the *BAT1* gene, and (C) with or without both modifications. A two-tailed Student’s t test was used to analyze the data statistically. Error bars: SEM for panels (A–C). (D–F) Effect of modulating the expression of genes involved in regulating icBCAA concentration on the lifespan defects of the K4A, K4R, or K121A cells. Statistical significance was determined using the Mann-Whitney test (∗*p* < 0.05, ∗∗*p* < 0.01, ∗∗∗*p* < 0.001).

Indeed, we found that the overexpression of *BAT2* partially reduced icBCAA levels in the H3K4A cells, and noticeably, the loss of *BAT1* significantly reduced icBCAA in H3K4A, H3K4R, and H3K121A strains (Figures 5A and 5B). Particularly in the H3K4A strain, *BAT1* deletion combined with *BAT2* overexpression additively lowered the high icBCAA levels (Figure 5C).

Consistently, we found that a combination of *bat1Δ* and *BAT2*-overexpression slightly extended the lifespan of the H3K4A strain (Figure 5D). Moreover, the simultaneous overexpression of both *BAT1* and *BAT2* partially reduced the lifespan defect of the H3K4R strain (Figure 5E). This rescue of lifespan in H3K4R is reminiscent of the addition of half a dose of BCAA (Figure 3H). We could not conclude that the lifespan defects caused by H3K4R or H3K121A were restored by *bat1Δ*, *BAT2*-overexpression, or their combination (Figures S7A and S7B).

In parallel, we found that the deletion of either *AGP1* or *BAP2* partially rescued the defective lifespan of the H3K121A strain (Figures 5F and S7C–S7E). Agp1 is a yeast amino acid permease involved in the uptake of Asn, Gln, and other amino acids.^34^ Bap2 is the high-affinity Leu permease involved in the uptake of Leu, Ile, and Val.^35^ Our result is in accordance with previous reports, which show that loss of either *AGP1* or *BAP2* leads to a decrease in BCAA uptake^35^^,^^36^ In addition, although *AGP1* and *BAP2* were downregulated to some degree by mutation on H3K4A/R and H3K121A, this level of downregulation appears insufficient to reduce the abnormally high icBCAA levels in these mutant cells.

### Epigenetic pathways of H3K4 or H3K121 on branched-chain amino acids-dependent lifespan include target of rapamycin complex 1 signaling

**Figure 6:**
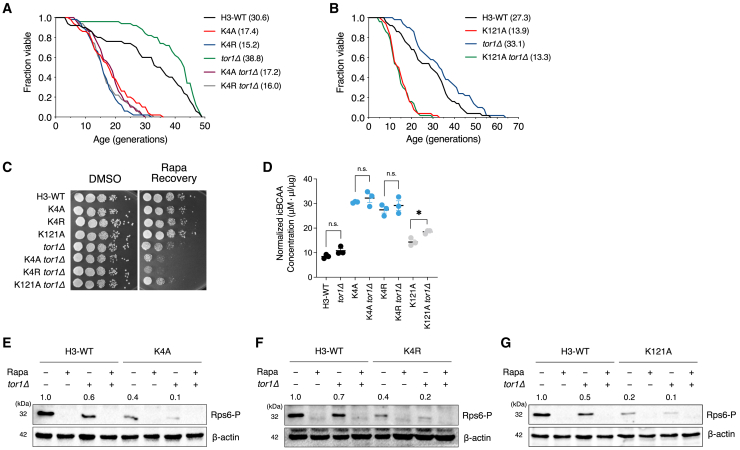
BCAA-dependent lifespan regulation through histone H3K4 or H3K121 includes the TORC1 signaling pathway (A and B) RLS analysis of the K4A, K4R, or K121A cells with or without *tor1Δ.* Statistical significance was determined using the Mann-Whitney test (∗*p* < 0.05, ∗∗*p* < 0.01, ∗∗∗*p* < 0.001). (C) Growth analysis of H3WT, H3K4A, H3K4R, and H3K121A strains with or without *tor1Δ.* Deletion of *TOR1* causes defects in growth recovery after the removal of 200 nM of rapamycin. (D) Quantifying free icBCAA levels in WT, H3K4A, H3K4R, H3K121A, and H3K121R cells with or without *tor1Δ.* Error bars: SEM for panels (D). (E–G) Western blot analyses of the phosphorylation of ribosomal protein-S6 (Rps6-p) in H3-WT, K4A (E), K4R (F), and K121A (G) cells with or without *tor1Δ.* Rapamycin was used to inhibit TORC1 pathways completely.

Next, we determined whether epigenetic modulation through H3K4 or H3K121 resulted in optimal cellular BCAA concentrations and whether such epigenetic regulation is associated with the TORC1 pathway in yeast. As reported earlier, cells deficient in *TOR1* significantly extended their lifespan (Figure 6A), ensuring the inhibition of TOR for longevity across eukaryotes.^37^ Intriguingly, we found that mutations at H3K4 or H3K121 suppressed the lifespan extension caused by TOR1 deletion, resulting in a lifespan similar to that of the single H3K4 or H3K121 mutants (Figures 6A and 6B), suggesting that BCAA-dependent lifespan regulation through H3K4 or H3K121 includes the TORC1 signaling pathway. Consistently, double mutants such as K4A *tor1Δ*, K4R *tor1Δ*, or K121A *tor1Δ* showed no or only a subtle effect on icBCAA levels (Figure 6D).

Additionally, mutations on H3K4 delayed recovery upon rapamycin treatment, which further explains the involvement of TORC1 pathways in H3K4-mediated regulation (Figure 6C). In parallel, mutations in H3K4A, H3K4R, or H3K121A significantly inhibited TORC1 activity to levels comparable to *tor1Δ* alone, and double mutations of *tor1Δ* combined with H3K4A, H3K4R or H3K121A inhibited TORC1 activity up to ground level, similarly by rapamycin treatment (Figures 6E–6G). Recently, it has been shown that BCAA metabolism is closely tied to TORC1 signaling,^27^ and we further show that the epigenetic regulation at histone H3K4 or H3K121, which maintains normal lifespan, involves TORC1 signaling pathways in yeast.

### Branched-chain amino acid metabolites partly rescue lifespan defects caused by mutations on H3K4 or H3K121

**Figure 7:**
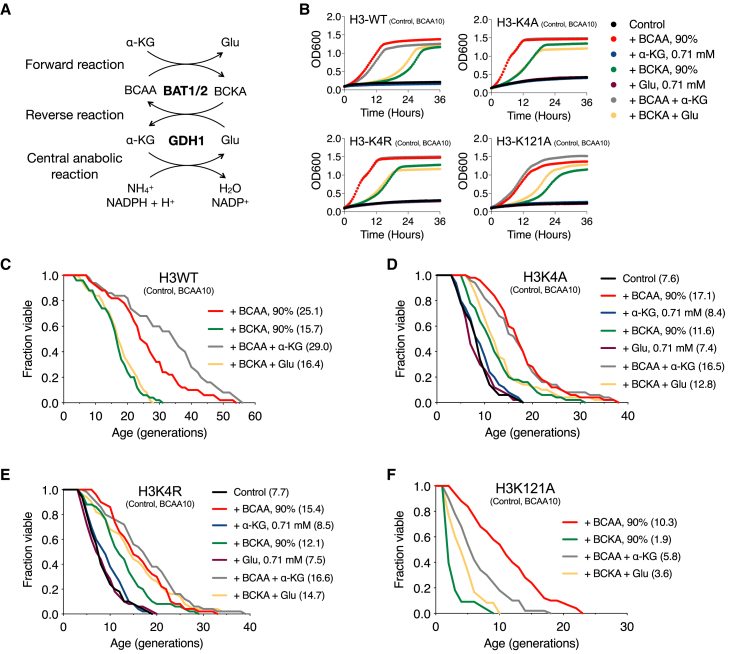
BCKA and other Bat1/2-mediated metabolites partly restore the alleviated replicative lifespan by mutations on H3K4 or H3K121 (A) The forward or reverse reaction of BCAA catabolism. (B) The growth analysis of WT, K4A, K4R, and K121A cells in SC medium with indicated supplementation; 90% of BCAA, 0.71 mM of α-KG, 90% of BCKA, 0.71 mM of glutamate, both 90% of BCAA and 0.71 mM of α-KG, or both 90% of BCKA and 0.71 mM of glutamate, respectively. Cells were supplemented with 10% BCAA, incubated up to 0.1 of OD_600_, and monitored for 36 h. The results represent mean values ±SEM from 3 independent experiments. (C–F) RLS analysis of WT (C), H3K4A (D), H3K4R (E), and H3K121A (F) on SC plate with indicated supplementation, as in Figure 6B. Statistical significance was determined using the Mann-Whitney test (∗*p* < 0.05, ∗∗*p* < 0.01, ∗∗∗*p* < 0.001).

The results so far provide evidence that epigenetic regulation through H3K4 or H3K121, which is required to maintain a normal lifespan, depends on BCAA homeostasis. This led us to examine whether BCAA metabolites resulting from the forward or reverse reaction of BCAA catabolism (also see Figure 7A)^38^ can rescue the impaired lifespan caused by mutations on H3K4 or H3K121. As seen in Figure 7B, cells showed minimal growth on media containing only 10% BCAA, but the addition of the remaining 90% fully restored their proliferation. Notably, branched-chain α-keto acids (BCKAs), metabolites generated via Bat1/2-mediated transamination, partially rescued the growth defect. In contrast, adding glutamate, one of the other Bat1/2 transamination products, or its reaction partner, α-ketoglutarate (α-KG), did not influence cell growth.

We then sought to determine whether the BCKA and other Bat1/2-mediated metabolites can also rescue the alleviated RLS by mutations on H3K4 or H3K121. Lifespan analysis could not be performed under BCAA10 control, α-KG, or glutamate conditions, as cells failed to divide (Figures 7C and 7F). Furthermore, the ability of H3K4A and H3K4R cells to divide even under 10% BCAA suggests that their elevated icBCAA levels were sufficient to meet the minimal requirement for cell division (Figures 7D and 7E. Also see Figure 3C).

Notably, the impaired lifespan caused by H3K4A, H3K4R, or H3K121A was partially restored by BCKA supplementation, similar to WT (Figures 7D–7F). This result is in line with the previous report that BCKAs are preferentially reaminated in the heart^39^ and suggests that the preferential reamination of BCKAs, rather than their oxidation, facilitates BCAA regeneration, which contributes to the partial recovery of lifespan. Either α-KG or glutamate sustained minimal cell division of H3K4A or H3K4R cells (Figures 7D and 7E), which is probably attributable to the role of Bat1/2 in H3K4-mediated lifespan regulation.

In the H3K121A strain, glutamate significantly extended its lifespan when co-supplemented with 90% BCKA. At the same time, α-KG unexpectedly suppressed the lifespan of the cells filled with optimal BCAA (Figure 7F). These results suggest that the H3K121A strain may rely on the reverse Bat1/2 reaction to restore lifespan, whereas WT cells benefit from the forward reaction. Lastly, although α-KG (0.71 mM) significantly increased the lifespan of the WT strain, increasing the concentration of α-KG paradoxically shortened the lifespan (Figures 7C and S8A). This longevity effect of α-KG (0.71 mM) was not seen in the H3K4A strain (Figure S8B), further suggesting that the H3K4A mutation limits the effect due to its high icBCAA level. These results show that epigenetic regulation at histones H3K4 or H3K121 is pivotal for maintaining intracellular BCAA homeostasis and a normal lifespan.

## Discussion

### A model proposes how the epigenetic modulations on H3K4 or H3K121 influence intracellular branched-chain amino acid levels to maintain a normal lifespan

**Figure 8:**
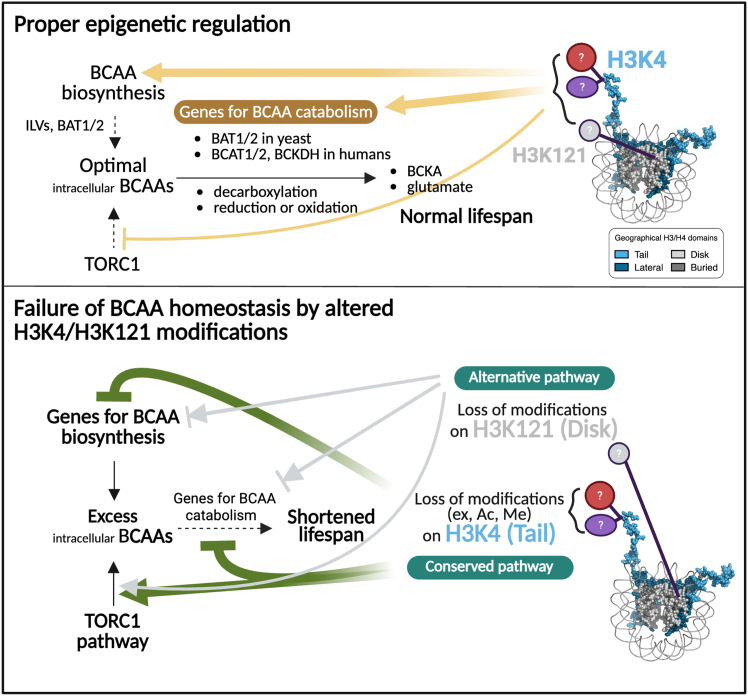
A proposed model for the epigenetic regulation of icBCAA homeostasis is required for a normal lifespan A possible epigenetic pathway that regulates icBCAA to maintain a normal lifespan is shown. See the discussion for details.

We propose an epigenetic pathway by which histone modifications regulate icBCAA levels to maintain a normal lifespan (Figure 8). Proper covalent modifications on histone H3 in WT cells, such as methylation or acetylation at K4 and potential modifications at K121, ensure optimal icBCAA levels by balancing BCAA biosynthesis and catabolism. Metabolites generated from BCAA turnover, including BCKAs and glutamate, are necessary to support normal lifespan. In addition, modifications at H3K4 in the tail domain and/or at H3K121 in the disk domain appear to restrict TOR activity; failure to do so may promote excessive protein synthesis and elevate icBCAA levels.

Abnormal elevation of icBCAAs is interpreted as a cellular “emergency state” that facilitates aging and shortens lifespan. This occurs through the loss of proper epigenetic information at lysine residues in the H3 tail or disk domains, particularly at K4 and K121, thereby disrupting the metabolic balance between BCAA biosynthesis and catabolism. Simultaneously, epigenetic dysregulation at these sites also perturbs TOR signaling. While K4 is evolutionarily conserved across eukaryotes, the K121 residue varies between species. Thus, H3K121 may serve a divergent or species-specific role in lifespan regulation, highlighting the uniqueness of our research, potentially unique to *S. cerevisiae*.

### Is P30 of histone H3 required for the methylation of K36?

The *cis*-trans isomerization of Pro in histone H3 regulates lysine methylation in *S. cerevisiae*, and especially, P38 isomerization by the proline isomerase Fpr4 is necessary for the methylation of K36.^21^ One of the long-lived strains screened in this study, P30A has the substitution at P30 of histone H3, which is located flanking P38 (see Figure 1C). Thus, we wondered if the extended lifespan by P30A substitution was due to the disruption of the K36 methylation. Although a report showed that the substitution of P30 to Val of histone H3 did not affect K36 methylation,^21^ it remains plausible that P30 is associated with K36 methylation: the isomerization of P30 is catalyzed by Fpr4 similarly to that of P38 *in vitro,*^21^ the *in vitro* methyltransferase activity on the synthetic peptides derived from the N-terminal sequence in H3 that contain known methylation sites was significantly decreased by P30 substitution to Ala,40 and loss of the Set2 methyltransferase extended lifespan.^40^ In addition, the substitution of P30 by Ala, but not by Val, is responsible for extending lifespan, raising the possible association between the isomerization of P38 and the methylation at K36 (Figure S1B).

The *cis*-trans isomerization of Pro in histone H3 regulates lysine methylation in *S. cerevisiae*, and especially, P38 isomerization by the proline isomerase Fpr4 is necessary for the methylation of K36.^21^ One of the LL strains screened in this study, P30A carries a substitution at P30 of histone H3, which is located adjacent to P38 (see Figure 1C). Thus, we wondered whether the extended lifespan conferred by the P30A substitution was due to the disruption of K36 methylation. However, H3P30A had little effect on H3 methylation at K36 (Figure S1B). Nevertheless, we do not exclude the possibility that P30 is associated with K36 methylation: isomerization of P30 is catalyzed by Fpr4 similarly to that of P38 *in vitro*^21^; *in vitro* methyltransferase activity on synthetic peptides derived from the N-terminal region of H3 containing known methylation sites was significantly decreased by the substitution of P30 with Ala^41^; loss of the Set2 methyltransferase extends lifespan^40^; and substitution of P30 with Ala itself is responsible for lifespan extension (Figure 1B).

An automated competition-based systematic assay for chronological lifespan in *S. cerevisiae* found *FPR4* to be one of the longevity factors, but it was not significant.^42^ In addition, the P30A substitution also had a marginal effect on the stability of histone H3 (Figure 1E). Therefore, these strongly suggest that a peptidyl-prolyl *cis*-trans isomerization at P30 of histone H3 may function as an anti-longevity epigenetic modification, probably through mechanisms independent of K36 methylation or histone stability, suggesting the involvement of previously unrecognized lifespan regulatory pathways.

### BCKDK, as a therapeutic for branched-chain amino acid-associated, H3K4 modification-defective human diseases?

The rate-limiting step of BCAA catabolism and clearance is the irreversible decarboxylation of BCAA-derived metabolites, BCKAs, by the branched-chain α-ketoacid dehydrogenase complex (BCKDH). Notably, the BCKDH is inhibited by the BCKDH kinase (BCKDK).^43^ Because deficiencies in BCAA catabolism are associated with insulin resistance, maple syrup urine disease, congenital heart disease, and heart failure,^12^ inhibition of BCKDK by the selective inhibitor 3,6-dichlorobenzo[*b*]thiophene-2-carboxylic acid (BT2) has emerged as a promising therapeutic approach for metabolic diseases and cancer in humans.^44^

The dysregulation of histone H3K4 methylations is associated with Alzheimer’s disease, carotid atherosclerosis, or carcinoma, such as esophageal squamous cell carcinomas.^45^^,^^46^^,^^47^ On the other hand, a study shows that histone acetylation at H3K4 by P300 plays an important role in regulating GATA4 expression in cardiogenesis.^48^ Additionally, genome-wide cell-based ChIP-Seq assays showed that H3K4 acetylation is associated with early stages of transformation^49^ while making gene enhancers in breast cancer cells.^50^ Thus, if there is an apparent elevation of plasma BCAA levels in patients with defects specifically related to H3K4 methylation or acetylation, it is possible that circulating plasma BCAAs and their catabolic products could be indicators of the onset of such neurodevelopmental or congenital heart disorders and the early stage of cancers. Therapeutics that selectively promote BCAA catabolism, such as BT2, could also be a potential target for such H3K4- and BCAA-associated disorders. However, we do not exclude the possibility that the epigenetic deregulation described in this study may have organism-wide effects and vary across different cell types.

### Limitations of the study

In this study, we primarily focused on screening histone point mutants using a comprehensive histone H3 and H4 substitution library to identify residues important for lifespan maintenance. Focusing now on the limitations of our approach, firstly, while we analyzed external RNA-seq datasets, including *set1Δ* mutants, to infer potential effects of methylation on H3K4, more detailed exploration would require experiments directly manipulating other histone-modifying enzymes such as acetyltransferases and deacetylases. Secondly, our experiments were conducted in budding yeast, and further investigations would be required to determine whether the epigenetic regulation of intracellular BCAA homeostasis identified here is conserved in higher eukaryotes. Lastly, although our findings implicate TORC1 signaling in lifespan regulation downstream of histone modifications, specific mechanistic experiments are required to clarify the causal relationship between histone modifications and TORC1 signaling pathways.

## Resource availability

### Lead contact

Further information and requests for resources and reagents should be directed to and will be fulfilled by the lead contact, Seong Hoon Ahn (hoon320@hanyang.ac.kr).

### Materials availability

• Yeast strains described in this study are available from the lead contact.
• This study did not generate new unique reagents.

### Data and code availability

• This study did not generate new datasets. RNA-seq datasets were downloaded from NCBI as follows: SRR12926606, SRR12926607, and SRR12926608 for K121A; SRR12926683, SRR12926684, SRR12926685, and SRR12926686 for the corresponding wild-type controls of the K121A data; SRR2517451, SRR2517456, and SRR2517461 for *set1Δ*; and SRR2517449, SRR2517454, and SRR2517459 for the corresponding wild-type controls of the *set1Δ* data. ATAC-seq datasets were downloaded from NCBI (SRA accession numbers: SRR13963722 and SRR13963723).
• This study does not report any original code.
• Any additional information required to reanalyze the data reported in this study is available from the lead contact upon request.

## Acknowledgments

This study was supported by the 10.13039/501100003725National Research Foundation of Korea (NRF) grant funded by the South Korean government (MSIT) [RS-2023-00243165 to S.H.A.]. We thank former lab members for the initial set-up of lifespan screening of histones and all members of the Ahn lab for their assistance in preparing the article. Schematics were created with BioRender.com.

## Author contributions

S.H.A. organized and designed the scope of the article and wrote the article with assistance from S.P. and Y.L. S.P. performed the geographical analysis of histones. S.P. and Y.L. performed the strain and/or plasmid construction used in this article. S.P., Y.L., and S.L. performed spotting assay and/or lifespan analysis. S.P. performed Western blot analysis. Y.L. performed measurements of the icBCAA assay and RT-PCR analysis. H.Y.R. and S.H.A. performed data analysis for all experiments.

## Declaration of interests

The authors declare no competing interests.

## STAR★Methods

### Key resources table

**Table:** 

REAGENT or RESOURCE	SOURCE	IDENTIFIER
**Antibodies**		

Anti-Histone H3	Abcam	Cat # ab1791; RRID: AB_302613
Anti-Histone H4	Abcam	Cat # ab5823; RRID: AB_10562795
Anti-Phospho-S6 Ribosomal Protein (Ser235/236)	Cell signaling	Cat # 2211; RRID: AB_331679
Anti-β-Actin	Abcam	Cat # ab8224; RRID: AB_449644
Anti-HA	Roche	Cat # 11583816001; RRID: AB_514505
Anti-H3K4me3	Abcam	Cat # ab8580; RRID: AB_3066649

**Bacterial and virus strains**		

*Escherichia coli* DH5a	N/A	N/A
*Saccharomyces cerevisiae*	N/A	N/A

**Chemicals, peptides, and recombinant proteins**		

Sulfometuron methyl (SM)	Sigma-Aldrich	Cat # 34224; CAS # 74222-97-2
Rapamycin	LC Laboratories	Cat # R-5000; CAS # 53123-88-9
Dimethyl sulfoxide (DMSO)	Sigma-Aldrich	Cat #D4540; CAS # 67-68-5

**Critical commercial assays**		

Branched Chain Amino Acid Assay Kit	Cell Biolabs	Cat # MET-5056
QIAprep spin miniprep kit	QIAGEN	Cat # 27106

**Deposited data**		

See Table S1	N/A	N/A
RNA-seq dataset	Braberg et al.^32^	SRA: SRR12926683, SRR12926684, SRR12926685, SRR12926686, SRR12926606, SRR12926607, SRR12926608
RNA-seq dataset	Chong et al.^33^	SRA: SRR2517449, SRR2517454 SRR2517459, SRR2517451, SRR2517456, SRR2517461
ATAC-seq dataset	Ramakrishnan et al.^31^	SRA: SRR13963722, SRR13963723

**Experimental models: Organisms/strains**		

*MATa ura3Δ0 leu2Δ0 trp1Δ63 his3Δ200 lys2Δ0 met15Δ0 can1::MFA1pr-HIS3 hht1-hhf1::NatMX4 hht2-hhf2::[HHTS-HHFS H3-WT]-URA3*	Open Biosystems	FY463
*MATa ura3Δ0 leu2Δ0 trp1Δ63 his3Δ200 lys2Δ0 met15Δ0 can1::MFA1pr-HIS3 hht1-hhf1::NatMX4 hht2-hhf2::[HHTS-HHFS H3A7S]-URA3*	Open Biosystems	FY471
*MATa ura3Δ0 leu2Δ0 trp1Δ63 his3Δ200 lys2Δ0 met15Δ0 can1::MFA1pr-HIS3 hht1-hhf1::NatMX4 hht2-hhf2::[HHTS-HHFS H3P30A]-URA3*	Open Biosystems	FY473
*MATa ura3Δ0 leu2Δ0 trp1Δ63 his3Δ200 lys2Δ0 met15Δ0 can1::MFA1pr-HIS3 hht1-hhf1::NatMX4 hht2-hhf2::[HHTS-HHFS H3A1S]-URA3*	Open Biosystems	FY663
*MATa ura3Δ0 leu2Δ0 trp1Δ63 his3Δ200 lys2Δ0 met15Δ0 can1::MFA1pr-HIS3 hht1-hhf1::NatMX4 hht2-hhf2::[HHTS-HHFS H3R2A]-URA3*	Open Biosystems	FY613
*MATa ura3Δ0 leu2Δ0 trp1Δ63 his3Δ200 lys2Δ0 met15Δ0 can1::MFA1pr-HIS3 hht1-hhf1::NatMX4 hht2-hhf2::[HHTS-HHFS H3R2K]-URA3*	Open Biosystems	FY650
*MATa ura3Δ0 leu2Δ0 trp1Δ63 his3Δ200 lys2Δ0 met15Δ0 can1::MFA1pr-HIS3 hht1-hhf1::NatMX4 hht2-hhf2::[HHTS-HHFS H3T3D]-URA3*	Open Biosystems	FY657
*MATa ura3Δ0 leu2Δ0 trp1Δ63 his3Δ200 lys2Δ0 met15Δ0 can1::MFA1pr-HIS3 hht1-hhf1::NatMX4 hht2-hhf2::[HHTS-HHFS H3K4A]-URA3*	Open Biosystems	FY811
*MATa ura3Δ0 leu2Δ0 trp1Δ63 his3Δ200 lys2Δ0 met15Δ0 can1::MFA1pr-HIS3 hht1-hhf1::NatMX4 hht2-hhf2::[HHTS-HHFS H3K4R]-URA3*	Open Biosystems	FY812
*MATa ura3Δ0 leu2Δ0 trp1Δ63 his3Δ200 lys2Δ0 met15Δ0 can1::MFA1pr-HIS3 hht1-hhf1::NatMX4 hht2-hhf2::[HHTS-HHFS H3K4Q]-URA3*	Open Biosystems	FY813
*MATa ura3Δ0 leu2Δ0 trp1Δ63 his3Δ200 lys2Δ0 met15Δ0 can1::MFA1pr-HIS3 hht1-hhf1::NatMX4 hht2-hhf2::[HHTS-HHFS H3Q5A]-URA3*	Open Biosystems	FY615
*MATa ura3Δ0 leu2Δ0 trp1Δ63 his3Δ200 lys2Δ0 met15Δ0 can1::MFA1pr-HIS3 hht1-hhf1::NatMX4 hht2-hhf2::[HHTS-HHFS H3Q5E]-URA3*	Open Biosystems	FY655
*MATa ura3Δ0 leu2Δ0 trp1Δ63 his3Δ200 lys2Δ0 met15Δ0 can1::MFA1pr-HIS3 hht1-hhf1::NatMX4 hht2-hhf2::[HHTS-HHFS H3R8K]-URA3*	Open Biosystems	FY651
*MATa ura3Δ0 leu2Δ0 trp1Δ63 his3Δ200 lys2Δ0 met15Δ0 can1::MFA1pr-HIS3 hht1-hhf1::NatMX4 hht2-hhf2::[HHTS-HHFS H3S10A]-URA3*	Open Biosystems	FY616
*MATa ura3Δ0 leu2Δ0 trp1Δ63 his3Δ200 lys2Δ0 met15Δ0 can1::MFA1pr-HIS3 hht1-hhf1::NatMX4 hht2-hhf2::[HHTS-HHFS H3T11D]-URA3*	Open Biosystems	FY658
*MATa ura3Δ0 leu2Δ0 trp1Δ63 his3Δ200 lys2Δ0 met15Δ0 can1::MFA1pr-HIS3 hht1-hhf1::NatMX4 hht2-hhf2::[HHTS-HHFS H3K14A]-URA3*	Open Biosystems	FY617
*MATa ura3Δ0 leu2Δ0 trp1Δ63 his3Δ200 lys2Δ0 met15Δ0 can1::MFA1pr-HIS3 hht1-hhf1::NatMX4 hht2-hhf2::[HHTS-HHFS H3K14Q]-URA3*	Open Biosystems	FY646
*MATa ura3Δ0 leu2Δ0 trp1Δ63 his3Δ200 lys2Δ0 met15Δ0 can1::MFA1pr-HIS3 hht1-hhf1::NatMX4 hht2-hhf2::[HHTS-HHFS H3R17K]-URA3*	Open Biosystems	FY652
*MATa ura3Δ0 leu2Δ0 trp1Δ63 his3Δ200 lys2Δ0 met15Δ0 can1::MFA1pr-HIS3 hht1-hhf1::NatMX4 hht2-hhf2::[HHTS-HHFS H3K18A]-URA3*	Open Biosystems	FY619
*MATa ura3Δ0 leu2Δ0 trp1Δ63 his3Δ200 lys2Δ0 met15Δ0 can1::MFA1pr-HIS3 hht1-hhf1::NatMX4 hht2-hhf2::[HHTS-HHFS H3K18Q]-URA3*	Open Biosystems	FY647
*MATa ura3Δ0 leu2Δ0 trp1Δ63 his3Δ200 lys2Δ0 met15Δ0 can1::MFA1pr-HIS3 hht1-hhf1::NatMX4 hht2-hhf2::[HHTS-HHFS H3L20A]-URA3*	Open Biosystems	FY620
*MATa ura3Δ0 leu2Δ0 trp1Δ63 his3Δ200 lys2Δ0 met15Δ0 can1::MFA1pr-HIS3 hht1-hhf1::NatMX4 hht2-hhf2::[HHTS-HHFS H3R26A]-URA3*	Open Biosystems	FY621
*MATa ura3Δ0 leu2Δ0 trp1Δ63 his3Δ200 lys2Δ0 met15Δ0 can1::MFA1pr-HIS3 hht1-hhf1::NatMX4 hht2-hhf2::[HHTS-HHFS H3K27R]-URA3*	Open Biosystems	FY472
*MATa ura3Δ0 leu2Δ0 trp1Δ63 his3Δ200 lys2Δ0 met15Δ0 can1::MFA1pr-HIS3 hht1-hhf1::NatMX4 hht2-hhf2::[HHTS-HHFS H3K36R]-URA3*	Open Biosystems	FY643
*MATa ura3Δ0 leu2Δ0 trp1Δ63 his3Δ200 lys2Δ0 met15Δ0 can1::MFA1pr-HIS3 hht1-hhf1::NatMX4 hht2-hhf2::[HHTS-HHFS H3K36Q]-URA3*	Open Biosystems	FY474
*MATa ura3Δ0 leu2Δ0 trp1Δ63 his3Δ200 lys2Δ0 met15Δ0 can1::MFA1pr-HIS3 hht1-hhf1::NatMX4 hht2-hhf2::[HHTS-HHFS H3K37Q]-URA3*	Open Biosystems	FY649
*MATa ura3Δ0 leu2Δ0 trp1Δ63 his3Δ200 lys2Δ0 met15Δ0 can1::MFA1pr-HIS3 hht1-hhf1::NatMX4 hht2-hhf2::[HHTS-HHFS H3P38V]-URA3*	Open Biosystems	FY475
*MATa ura3Δ0 leu2Δ0 trp1Δ63 his3Δ200 lys2Δ0 met15Δ0 can1::MFA1pr-HIS3 hht1-hhf1::NatMX4 hht2-hhf2::[HHTS-HHFS H3K42A]-URA3*	Open Biosystems	FY624
*MATa ura3Δ0 leu2Δ0 trp1Δ63 his3Δ200 lys2Δ0 met15Δ0 can1::MFA1pr-HIS3 hht1-hhf1::NatMX4 hht2-hhf2::[HHTS-HHFS H3K42Q]-URA3*	Open Biosystems	FY476
*MATa ura3Δ0 leu2Δ0 trp1Δ63 his3Δ200 lys2Δ0 met15Δ0 can1::MFA1pr-HIS3 hht1-hhf1::NatMX4 hht2-hhf2::[HHTS-HHFS H3G44A]-URA3*	Open Biosystems	FY477
*MATa ura3Δ0 leu2Δ0 trp1Δ63 his3Δ200 lys2Δ0 met15Δ0 can1::MFA1pr-HIS3 hht1-hhf1::NatMX4 hht2-hhf2::[HHTS-HHFS H3V46A]-URA3*	Open Biosystems	FY625
*MATa ura3Δ0 leu2Δ0 trp1Δ63 his3Δ200 lys2Δ0 met15Δ0 can1::MFA1pr-HIS3 hht1-hhf1::NatMX4 hht2-hhf2::[HHTS-HHFS H3R49A]-URA3*	Open Biosystems	FY478
*MATa ura3Δ0 leu2Δ0 trp1Δ63 his3Δ200 lys2Δ0 met15Δ0 can1::MFA1pr-HIS3 hht1-hhf1::NatMX4 hht2-hhf2::[HHTS-HHFS H3R49K]-URA3*	Open Biosystems	FY479
*MATa ura3Δ0 leu2Δ0 trp1Δ63 his3Δ200 lys2Δ0 met15Δ0 can1::MFA1pr-HIS3 hht1-hhf1::NatMX4 hht2-hhf2::[HHTS-HHFS H3K53A]-URA3*	Open Biosystems	FY626
*MATa ura3Δ0 leu2Δ0 trp1Δ63 his3Δ200 lys2Δ0 met15Δ0 can1::MFA1pr-HIS3 hht1-hhf1::NatMX4 hht2-hhf2::[HHTS-HHFS H3K56Q]-URA3*	Open Biosystems	FY482
*MATa ura3Δ0 leu2Δ0 trp1Δ63 his3Δ200 lys2Δ0 met15Δ0 can1::MFA1pr-HIS3 hht1-hhf1::NatMX4 hht2-hhf2::[HHTS-HHFS H3S57D]-URA3*	Open Biosystems	FY483
*MATa ura3Δ0 leu2Δ0 trp1Δ63 his3Δ200 lys2Δ0 met15Δ0 can1::MFA1pr-HIS3 hht1-hhf1::NatMX4 hht2-hhf2::[HHTS-HHFS H3E59Q]-URA3*	Open Biosystems	FY654
*MATa ura3Δ0 leu2Δ0 trp1Δ63 his3Δ200 lys2Δ0 met15Δ0 can1::MFA1pr-HIS3 hht1-hhf1::NatMX4 hht2-hhf2::[HHTS-HHFS H3L61A]-URA3*	Open Biosystems	FY488
*MATa ura3Δ0 leu2Δ0 trp1Δ63 his3Δ200 lys2Δ0 met15Δ0 can1::MFA1pr-HIS3 hht1-hhf1::NatMX4 hht2-hhf2::[HHTS-HHFS H3K64Q]-URA3*	Open Biosystems	FY484
*MATa ura3Δ0 leu2Δ0 trp1Δ63 his3Δ200 lys2Δ0 met15Δ0 can1::MFA1pr-HIS3 hht1-hhf1::NatMX4 hht2-hhf2::[HHTS-HHFS H3Q68A]-URA3*	Open Biosystems	FY485
*MATa ura3Δ0 leu2Δ0 trp1Δ63 his3Δ200 lys2Δ0 met15Δ0 can1::MFA1pr-HIS3 hht1-hhf1::NatMX4 hht2-hhf2::[HHTS-HHFS H3R69A]-URA3*	Open Biosystems	FY627
*MATa ura3Δ0 leu2Δ0 trp1Δ63 his3Δ200 lys2Δ0 met15Δ0 can1::MFA1pr-HIS3 hht1-hhf1::NatMX4 hht2-hhf2::[HHTS-HHFS H3R69K]-URA3*	Open Biosystems	FY486
*MATa ura3Δ0 leu2Δ0 trp1Δ63 his3Δ200 lys2Δ0 met15Δ0 can1::MFA1pr-HIS3 hht1-hhf1::NatMX4 hht2-hhf2::[HHTS-HHFS H3L70A]-URA3*	Open Biosystems	FY628
*MATa ura3Δ0 leu2Δ0 trp1Δ63 his3Δ200 lys2Δ0 met15Δ0 can1::MFA1pr-HIS3 hht1-hhf1::NatMX4 hht2-hhf2::[HHTS-HHFS H3E73A]-URA3*	Open Biosystems	FY487
*MATa ura3Δ0 leu2Δ0 trp1Δ63 his3Δ200 lys2Δ0 met15Δ0 can1::MFA1pr-HIS3 hht1-hhf1::NatMX4 hht2-hhf2::[HHTS-HHFS H3D77N]-URA3*	Open Biosystems	FY653
*MATa ura3Δ0 leu2Δ0 trp1Δ63 his3Δ200 lys2Δ0 met15Δ0 can1::MFA1pr-HIS3 hht1-hhf1::NatMX4 hht2-hhf2::[HHTS-HHFS H3F78A]-URA3*	Open Biosystems	FY629
*MATa ura3Δ0 leu2Δ0 trp1Δ63 his3Δ200 lys2Δ0 met15Δ0 can1::MFA1pr-HIS3 hht1-hhf1::NatMX4 hht2-hhf2::[HHTS-HHFS H3K79A]-URA3*	Open Biosystems	FY630
*MATa ura3Δ0 leu2Δ0 trp1Δ63 his3Δ200 lys2Δ0 met15Δ0 can1::MFA1pr-HIS3 hht1-hhf1::NatMX4 hht2-hhf2::[HHTS-HHFS H3K79R]-URA3*	Open Biosystems	FY644
*MATa ura3Δ0 leu2Δ0 trp1Δ63 his3Δ200 lys2Δ0 met15Δ0 can1::MFA1pr-HIS3 hht1-hhf1::NatMX4 hht2-hhf2::[HHTS-HHFS H3T80D]-URA3*	Open Biosystems	FY660
*MATa ura3Δ0 leu2Δ0 trp1Δ63 his3Δ200 lys2Δ0 met15Δ0 can1::MFA1pr-HIS3 hht1-hhf1::NatMX4 hht2-hhf2::[HHTS-HHFS H3L82A]-URA3*	Open Biosystems	FY631
*MATa ura3Δ0 leu2Δ0 trp1Δ63 his3Δ200 lys2Δ0 met15Δ0 can1::MFA1pr-HIS3 hht1-hhf1::NatMX4 hht2-hhf2::[HHTS-HHFS H3R83A]-URA3*	Open Biosystems	FY632
*MATa ura3Δ0 leu2Δ0 trp1Δ63 his3Δ200 lys2Δ0 met15Δ0 can1::MFA1pr-HIS3 hht1-hhf1::NatMX4 hht2-hhf2::[HHTS-HHFS H3Q85A]-URA3*	Open Biosystems	FY633
*MATa ura3Δ0 leu2Δ0 trp1Δ63 his3Δ200 lys2Δ0 met15Δ0 can1::MFA1pr-HIS3 hht1-hhf1::NatMX4 hht2-hhf2::[HHTS-HHFS H3S86A]-URA3*	Open Biosystems	FY489
*MATa ura3Δ0 leu2Δ0 trp1Δ63 his3Δ200 lys2Δ0 met15Δ0 can1::MFA1pr-HIS3 hht1-hhf1::NatMX4 hht2-hhf2::[HHTS-HHFS H3S86D]-URA3*	Open Biosystems	FY490
*MATa ura3Δ0 leu2Δ0 trp1Δ63 his3Δ200 lys2Δ0 met15Δ0 can1::MFA1pr-HIS3 hht1-hhf1::NatMX4 hht2-hhf2::[HHTS-HHFS H3S87A]-URA3*	Open Biosystems	FY634
*MATa ura3Δ0 leu2Δ0 trp1Δ63 his3Δ200 lys2Δ0 met15Δ0 can1::MFA1pr-HIS3 hht1-hhf1::NatMX4 hht2-hhf2::[HHTS-HHFS H3S87D]-URA3*	Open Biosystems	FY491
*MATa ura3Δ0 leu2Δ0 trp1Δ63 his3Δ200 lys2Δ0 met15Δ0 can1::MFA1pr-HIS3 hht1-hhf1::NatMX4 hht2-hhf2::[HHTS-HHFS H3I89A]-URA3*	Open Biosystems	FY492
*MATa ura3Δ0 leu2Δ0 trp1Δ63 his3Δ200 lys2Δ0 met15Δ0 can1::MFA1pr-HIS3 hht1-hhf1::NatMX4 hht2-hhf2::[HHTS-HHFS H3G90A]-URA3*	Open Biosystems	FY493
*MATa ura3Δ0 leu2Δ0 trp1Δ63 his3Δ200 lys2Δ0 met15Δ0 can1::MFA1pr-HIS3 hht1-hhf1::NatMX4 hht2-hhf2::[HHTS-HHFS H3Y99A]-URA3*	Open Biosystems	FY494
*MATa ura3Δ0 leu2Δ0 trp1Δ63 his3Δ200 lys2Δ0 met15Δ0 can1::MFA1pr-HIS3 hht1-hhf1::NatMX4 hht2-hhf2::[HHTS-HHFS H3V101A]-URA3*	Open Biosystems	FY495
*MATa ura3Δ0 leu2Δ0 trp1Δ63 his3Δ200 lys2Δ0 met15Δ0 can1::MFA1pr-HIS3 hht1-hhf1::NatMX4 hht2-hhf2::[HHTS-HHFS H3F104A]-URA3*	Open Biosystems	FY496
*MATa ura3Δ0 leu2Δ0 trp1Δ63 his3Δ200 lys2Δ0 met15Δ0 can1::MFA1pr-HIS3 hht1-hhf1::NatMX4 hht2-hhf2::[HHTS-HHFS H3E105Q]-URA3*	Open Biosystems	FY497
*MATa ura3Δ0 leu2Δ0 trp1Δ63 his3Δ200 lys2Δ0 met15Δ0 can1::MFA1pr-HIS3 hht1-hhf1::NatMX4 hht2-hhf2::[HHTS-HHFS H3D106A]-URA3*	Open Biosystems	FY635
*MATa ura3Δ0 leu2Δ0 trp1Δ63 his3Δ200 lys2Δ0 met15Δ0 can1::MFA1pr-HIS3 hht1-hhf1::NatMX4 hht2-hhf2::[HHTS-HHFS H3N108A]-URA3*	Open Biosystems	FY499
*MATa ura3Δ0 leu2Δ0 trp1Δ63 his3Δ200 lys2Δ0 met15Δ0 can1::MFA1pr-HIS3 hht1-hhf1::NatMX4 hht2-hhf2::[HHTS-HHFS H3A114S]-URA3*	Open Biosystems	FY636
*MATa ura3Δ0 leu2Δ0 trp1Δ63 his3Δ200 lys2Δ0 met15Δ0 can1::MFA1pr-HIS3 hht1-hhf1::NatMX4 hht2-hhf2::[HHTS-HHFS H3V117A]-URA3*	Open Biosystems	FY500
*MATa ura3Δ0 leu2Δ0 trp1Δ63 his3Δ200 lys2Δ0 met15Δ0 can1::MFA1pr-HIS3 hht1-hhf1::NatMX4 hht2-hhf2::[HHTS-HHFS H3Q120A]-URA3*	Open Biosystems	FY637
*MATa ura3Δ0 leu2Δ0 trp1Δ63 his3Δ200 lys2Δ0 met15Δ0 can1::MFA1pr-HIS3 hht1-hhf1::NatMX4 hht2-hhf2::[HHTS-HHFS H3K121A]-URA3*	Open Biosystems	FY501
*MATa ura3Δ0 leu2Δ0 trp1Δ63 his3Δ200 lys2Δ0 met15Δ0 can1::MFA1pr-HIS3 hht1-hhf1::NatMX4 hht2-hhf2::[HHTS-HHFS H3K121R]-URA3*	Open Biosystems	FY645
*MATa ura3Δ0 leu2Δ0 trp1Δ63 his3Δ200 lys2Δ0 met15Δ0 can1::MFA1pr-HIS3 hht1-hhf1::NatMX4 hht2-hhf2::[HHTS-HHFS H3K122R]-URA3*	Open Biosystems	FY502
*MATa ura3Δ0 leu2Δ0 trp1Δ63 his3Δ200 lys2Δ0 met15Δ0 can1::MFA1pr-HIS3 hht1-hhf1::NatMX4 hht2-hhf2::[HHTS-HHFS H3K125Q]-URA3*	Open Biosystems	FY503
*MATa ura3Δ0 leu2Δ0 trp1Δ63 his3Δ200 lys2Δ0 met15Δ0 can1::MFA1pr-HIS3 hht1-hhf1::NatMX4 hht2-hhf2::[HHTS-HHFS H3R129A]-URA3*	Open Biosystems	FY639
*MATa ura3Δ0 leu2Δ0 trp1Δ63 his3Δ200 lys2Δ0 met15Δ0 can1::MFA1pr-HIS3 hht1-hhf1::NatMX4 hht2-hhf2::[HHTS-HHFS H3R131A]-URA3*	Open Biosystems	FY640
*MATa ura3Δ0 leu2Δ0 trp1Δ63 his3Δ200 lys2Δ0 met15Δ0 can1::MFA1pr-HIS3 hht1-hhf1::NatMX4 hht2-hhf2::[HHTS-HHFS H3G132A]-URA3*	Open Biosystems	FY641
*MATa ura3Δ0 leu2Δ0 trp1Δ63 his3Δ200 lys2Δ0 met15Δ0 can1::MFA1pr-HIS3 hht1-hhf1::NatMX4 hht2-hhf2::[HHTS-HHFS H3E133Q]-URA3*	Open Biosystems	FY504
*MATa ura3Δ0 leu2Δ0 trp1Δ63 his3Δ200 lys2Δ0 met15Δ0 can1::MFA1pr-HIS3 hht1-hhf1::NatMX4 hht2-hhf2::[HHTS-HHFS H3S135A]-URA3*	Open Biosystems	FY505
*MATa ura3Δ0 leu2Δ0 trp1Δ63 his3Δ200 lys2Δ0 met15Δ0 can1::MFA1pr-HIS3 hht1-hhf1::NatMX4 hht2-hhf2::[HHTS-HHFS H4-WT]-URA3*	Open Biosystems	FY506
*MATa ura3Δ0 leu2Δ0 trp1Δ63 his3Δ200 lys2Δ0 met15Δ0 can1::MFA1pr-HIS3 hht1-hhf1::NatMX4 hht2-hhf2::[HHTS-HHFS H4S1A]-URA3*	Open Biosystems	FY664
*MATa ura3Δ0 leu2Δ0 trp1Δ63 his3Δ200 lys2Δ0 met15Δ0 can1::MFA1pr-HIS3 hht1-hhf1::NatMX4 hht2-hhf2::[HHTS-HHFS H4G2A]-URA3*	Open Biosystems	FY665
*MATa ura3Δ0 leu2Δ0 trp1Δ63 his3Δ200 lys2Δ0 met15Δ0 can1::MFA1pr-HIS3 hht1-hhf1::NatMX4 hht2-hhf2::[HHTS-HHFS H4R3K]-URA3*	Open Biosystems	FY536
*MATa ura3Δ0 leu2Δ0 trp1Δ63 his3Δ200 lys2Δ0 met15Δ0 can1::MFA1pr-HIS3 hht1-hhf1::NatMX4 hht2-hhf2::[HHTS-HHFS H4K5A]-URA3*	Open Biosystems	FY523
*MATa ura3Δ0 leu2Δ0 trp1Δ63 his3Δ200 lys2Δ0 met15Δ0 can1::MFA1pr-HIS3 hht1-hhf1::NatMX4 hht2-hhf2::[HHTS-HHFS H4K8A]-URA3*	Open Biosystems	FY526
*MATa ura3Δ0 leu2Δ0 trp1Δ63 his3Δ200 lys2Δ0 met15Δ0 can1::MFA1pr-HIS3 hht1-hhf1::NatMX4 hht2-hhf2::[HHTS-HHFS H4G11A]-URA3*	Open Biosystems	FY668
*MATa ura3Δ0 leu2Δ0 trp1Δ63 his3Δ200 lys2Δ0 met15Δ0 can1::MFA1pr-HIS3 hht1-hhf1::NatMX4 hht2-hhf2::[HHTS-HHFS H4G14A]-URA3*	Open Biosystems	FY670
*MATa ura3Δ0 leu2Δ0 trp1Δ63 his3Δ200 lys2Δ0 met15Δ0 can1::MFA1pr-HIS3 hht1-hhf1::NatMX4 hht2-hhf2::[HHTS-HHFS H4K16A]-URA3*	Open Biosystems	FY532
*MATa ura3Δ0 leu2Δ0 trp1Δ63 his3Δ200 lys2Δ0 met15Δ0 can1::MFA1pr-HIS3 hht1-hhf1::NatMX4 hht2-hhf2::[HHTS-HHFS H4R17K]-URA3*	Open Biosystems	FY715
*MATa ura3Δ0 leu2Δ0 trp1Δ63 his3Δ200 lys2Δ0 met15Δ0 can1::MFA1pr-HIS3 hht1-hhf1::NatMX4 hht2-hhf2::[HHTS-HHFS H4H18A]-URA3*	Open Biosystems	FY673
*MATa ura3Δ0 leu2Δ0 trp1Δ63 his3Δ200 lys2Δ0 met15Δ0 can1::MFA1pr-HIS3 hht1-hhf1::NatMX4 hht2-hhf2::[HHTS-HHFS H4R19A]-URA3*	Open Biosystems	FY674
*MATa ura3Δ0 leu2Δ0 trp1Δ63 his3Δ200 lys2Δ0 met15Δ0 can1::MFA1pr-HIS3 hht1-hhf1::NatMX4 hht2-hhf2::[HHTS-HHFS H4R23A]-URA3*	Open Biosystems	FY675
*MATa ura3Δ0 leu2Δ0 trp1Δ63 his3Δ200 lys2Δ0 met15Δ0 can1::MFA1pr-HIS3 hht1-hhf1::NatMX4 hht2-hhf2::[HHTS-HHFS H4D24N]-URA3*	Open Biosystems	FY723
*MATa ura3Δ0 leu2Δ0 trp1Δ63 his3Δ200 lys2Δ0 met15Δ0 can1::MFA1pr-HIS3 hht1-hhf1::NatMX4 hht2-hhf2::[HHTS-HHFS H4N25A]-URA3*	Open Biosystems	FY676
*MATa ura3Δ0 leu2Δ0 trp1Δ63 his3Δ200 lys2Δ0 met15Δ0 can1::MFA1pr-HIS3 hht1-hhf1::NatMX4 hht2-hhf2::[HHTS-HHFS H4I29A]-URA3*	Open Biosystems	FY677
*MATa ura3Δ0 leu2Δ0 trp1Δ63 his3Δ200 lys2Δ0 met15Δ0 can1::MFA1pr-HIS3 hht1-hhf1::NatMX4 hht2-hhf2::[HHTS-HHFS H4P32A]-URA3*	Open Biosystems	FY678
*MATa ura3Δ0 leu2Δ0 trp1Δ63 his3Δ200 lys2Δ0 met15Δ0 can1::MFA1pr-HIS3 hht1-hhf1::NatMX4 hht2-hhf2::[HHTS-HHFS H4A33S]-URA3*	Open Biosystems	FY679
*MATa ura3Δ0 leu2Δ0 trp1Δ63 his3Δ200 lys2Δ0 met15Δ0 can1::MFA1pr-HIS3 hht1-hhf1::NatMX4 hht2-hhf2::[HHTS-HHFS H4R36A]-URA3*	Open Biosystems	FY680
*MATa ura3Δ0 leu2Δ0 trp1Δ63 his3Δ200 lys2Δ0 met15Δ0 can1::MFA1pr-HIS3 hht1-hhf1::NatMX4 hht2-hhf2::[HHTS-HHFS H4R36K]-URA3*	Open Biosystems	FY717
*MATa ura3Δ0 leu2Δ0 trp1Δ63 his3Δ200 lys2Δ0 met15Δ0 can1::MFA1pr-HIS3 hht1-hhf1::NatMX4 hht2-hhf2::[HHTS-HHFS H4R39K]-URA3*	Open Biosystems	FY718
*MATa ura3Δ0 leu2Δ0 trp1Δ63 his3Δ200 lys2Δ0 met15Δ0 can1::MFA1pr-HIS3 hht1-hhf1::NatMX4 hht2-hhf2::[HHTS-HHFS H4R40K]-URA3*	Open Biosystems	FY719
*MATa ura3Δ0 leu2Δ0 trp1Δ63 his3Δ200 lys2Δ0 met15Δ0 can1::MFA1pr-HIS3 hht1-hhf1::NatMX4 hht2-hhf2::[HHTS-HHFS H4V43A]-URA3*	Open Biosystems	FY681
*MATa ura3Δ0 leu2Δ0 trp1Δ63 his3Δ200 lys2Δ0 met15Δ0 can1::MFA1pr-HIS3 hht1-hhf1::NatMX4 hht2-hhf2::[HHTS-HHFS H4K44A]-URA3*	Open Biosystems	FY682
*MATa ura3Δ0 leu2Δ0 trp1Δ63 his3Δ200 lys2Δ0 met15Δ0 can1::MFA1pr-HIS3 hht1-hhf1::NatMX4 hht2-hhf2::[HHTS-HHFS H4K44R]-URA3*	Open Biosystems	FY707
*MATa ura3Δ0 leu2Δ0 trp1Δ63 his3Δ200 lys2Δ0 met15Δ0 can1::MFA1pr-HIS3 hht1-hhf1::NatMX4 hht2-hhf2::[HHTS-HHFS H4S47D]-URA3*	Open Biosystems	FY725
*MATa ura3Δ0 leu2Δ0 trp1Δ63 his3Δ200 lys2Δ0 met15Δ0 can1::MFA1pr-HIS3 hht1-hhf1::NatMX4 hht2-hhf2::[HHTS-HHFS H4I50A]-URA3*	Open Biosystems	FY684
*MATa ura3Δ0 leu2Δ0 trp1Δ63 his3Δ200 lys2Δ0 met15Δ0 can1::MFA1pr-HIS3 hht1-hhf1::NatMX4 hht2-hhf2::[HHTS-HHFS H4E53Q]-URA3*	Open Biosystems	FY722
*MATa ura3Δ0 leu2Δ0 trp1Δ63 his3Δ200 lys2Δ0 met15Δ0 can1::MFA1pr-HIS3 hht1-hhf1::NatMX4 hht2-hhf2::[HHTS-HHFS H4V54A]-URA3*	Open Biosystems	FY685
*MATa ura3Δ0 leu2Δ0 trp1Δ63 his3Δ200 lys2Δ0 met15Δ0 can1::MFA1pr-HIS3 hht1-hhf1::NatMX4 hht2-hhf2::[HHTS-HHFS H4L58A]-URA3*	Open Biosystems	FY686
*MATa ura3Δ0 leu2Δ0 trp1Δ63 his3Δ200 lys2Δ0 met15Δ0 can1::MFA1pr-HIS3 hht1-hhf1::NatMX4 hht2-hhf2::[HHTS-HHFS H4K59Q]-URA3*	Open Biosystems	FY726
*MATa ura3Δ0 leu2Δ0 trp1Δ63 his3Δ200 lys2Δ0 met15Δ0 can1::MFA1pr-HIS3 hht1-hhf1::NatMX4 hht2-hhf2::[HHTS-HHFS H4L62A]-URA3*	Open Biosystems	FY687
*MATa ura3Δ0 leu2Δ0 trp1Δ63 his3Δ200 lys2Δ0 met15Δ0 can1::MFA1pr-HIS3 hht1-hhf1::NatMX4 hht2-hhf2::[HHTS-HHFS H4I66A]-URA3*	Open Biosystems	FY688
*MATa ura3Δ0 leu2Δ0 trp1Δ63 his3Δ200 lys2Δ0 met15Δ0 can1::MFA1pr-HIS3 hht1-hhf1::NatMX4 hht2-hhf2::[HHTS-HHFS H4R67A]-URA3*	Open Biosystems	FY689
*MATa ura3Δ0 leu2Δ0 trp1Δ63 his3Δ200 lys2Δ0 met15Δ0 can1::MFA1pr-HIS3 hht1-hhf1::NatMX4 hht2-hhf2::[HHTS-HHFS H4D68A]-URA3*	Open Biosystems	FY690
*MATa ura3Δ0 leu2Δ0 trp1Δ63 his3Δ200 lys2Δ0 met15Δ0 can1::MFA1pr-HIS3 hht1-hhf1::NatMX4 hht2-hhf2::[HHTS-HHFS H4V70A]-URA3*	Open Biosystems	FY691
*MATa ura3Δ0 leu2Δ0 trp1Δ63 his3Δ200 lys2Δ0 met15Δ0 can1::MFA1pr-HIS3 hht1-hhf1::NatMX4 hht2-hhf2::[HHTS-HHFS H4E74A]-URA3*	Open Biosystems	FY692
*MATa ura3Δ0 leu2Δ0 trp1Δ63 his3Δ200 lys2Δ0 met15Δ0 can1::MFA1pr-HIS3 hht1-hhf1::NatMX4 hht2-hhf2::[HHTS-HHFS H4H75Q]-URA3*	Open Biosystems	FY528
*MATa ura3Δ0 leu2Δ0 trp1Δ63 his3Δ200 lys2Δ0 met15Δ0 can1::MFA1pr-HIS3 hht1-hhf1::NatMX4 hht2-hhf2::[HHTS-HHFS H4R78K]-URA3*	Open Biosystems	FY720
*MATa ura3Δ0 leu2Δ0 trp1Δ63 his3Δ200 lys2Δ0 met15Δ0 can1::MFA1pr-HIS3 hht1-hhf1::NatMX4 hht2-hhf2::[HHTS-HHFS H4K79A]-URA3*	Open Biosystems	FY693
*MATa ura3Δ0 leu2Δ0 trp1Δ63 his3Δ200 lys2Δ0 met15Δ0 can1::MFA1pr-HIS3 hht1-hhf1::NatMX4 hht2-hhf2::[HHTS-HHFS H4K79R]-URA3*	Open Biosystems	FY708
*MATa ura3Δ0 leu2Δ0 trp1Δ63 his3Δ200 lys2Δ0 met15Δ0 can1::MFA1pr-HIS3 hht1-hhf1::NatMX4 hht2-hhf2::[HHTS-HHFS H4K79Q]-URA3*	Open Biosystems	FY713
*MATa ura3Δ0 leu2Δ0 trp1Δ63 his3Δ200 lys2Δ0 met15Δ0 can1::MFA1pr-HIS3 hht1-hhf1::NatMX4 hht2-hhf2::[HHTS-HHFS H4T80A]-URA3*	Open Biosystems	FY694
*MATa ura3Δ0 leu2Δ0 trp1Δ63 his3Δ200 lys2Δ0 met15Δ0 can1::MFA1pr-HIS3 hht1-hhf1::NatMX4 hht2-hhf2::[HHTS-HHFS H4V81A]-URA3*	Open Biosystems	FY695
*MATa ura3Δ0 leu2Δ0 trp1Δ63 his3Δ200 lys2Δ0 met15Δ0 can1::MFA1pr-HIS3 hht1-hhf1::NatMX4 hht2-hhf2::[HHTS-HHFS H4S83A]-URA3*	Open Biosystems	FY696
*MATa ura3Δ0 leu2Δ0 trp1Δ63 his3Δ200 lys2Δ0 met15Δ0 can1::MFA1pr-HIS3 hht1-hhf1::NatMX4 hht2-hhf2::[HHTS-HHFS H4D85A]-URA3*	Open Biosystems	FY697
*MATa ura3Δ0 leu2Δ0 trp1Δ63 his3Δ200 lys2Δ0 met15Δ0 can1::MFA1pr-HIS3 hht1-hhf1::NatMX4 hht2-hhf2::[HHTS-HHFS H4V86A]-URA3*	Open Biosystems	FY698
*MATa ura3Δ0 leu2Δ0 trp1Δ63 his3Δ200 lys2Δ0 met15Δ0 can1::MFA1pr-HIS3 hht1-hhf1::NatMX4 hht2-hhf2::[HHTS-HHFS H4Y88A]-URA3*	Open Biosystems	FY699
*MATa ura3Δ0 leu2Δ0 trp1Δ63 his3Δ200 lys2Δ0 met15Δ0 can1::MFA1pr-HIS3 hht1-hhf1::NatMX4 hht2-hhf2::[HHTS-HHFS H4Y88F]-URA3*	Open Biosystems	FY727
*MATa ura3Δ0 leu2Δ0 trp1Δ63 his3Δ200 lys2Δ0 met15Δ0 can1::MFA1pr-HIS3 hht1-hhf1::NatMX4 hht2-hhf2::[HHTS-HHFS H4K91R]-URA3*	Open Biosystems	FY538
*MATa ura3Δ0 leu2Δ0 trp1Δ63 his3Δ200 lys2Δ0 met15Δ0 can1::MFA1pr-HIS3 hht1-hhf1::NatMX4 hht2-hhf2::[HHTS-HHFS H4R92K]-URA3*	Open Biosystems	FY716
*MATa ura3Δ0 leu2Δ0 trp1Δ63 his3Δ200 lys2Δ0 met15Δ0 can1::MFA1pr-HIS3 hht1-hhf1::NatMX4 hht2-hhf2::[HHTS-HHFS H4Q93A]-URA3*	Open Biosystems	FY700
*MATa ura3Δ0 leu2Δ0 trp1Δ63 his3Δ200 lys2Δ0 met15Δ0 can1::MFA1pr-HIS3 hht1-hhf1::NatMX4 hht2-hhf2::[HHTS-HHFS H4Q93E]-URA3*	Open Biosystems	FY721
*MATa ura3Δ0 leu2Δ0 trp1Δ63 his3Δ200 lys2Δ0 met15Δ0 can1::MFA1pr-HIS3 hht1-hhf1::NatMX4 hht2-hhf2::[HHTS-HHFS H4G94A]-URA3*	Open Biosystems	FY701
*MATa ura3Δ0 leu2Δ0 trp1Δ63 his3Δ200 lys2Δ0 met15Δ0 can1::MFA1pr-HIS3 hht1-hhf1::NatMX4 hht2-hhf2::[HHTS-HHFS H4T96A]-URA3*	Open Biosystems	FY702
*MATa ura3Δ0 leu2Δ0 trp1Δ63 his3Δ200 lys2Δ0 met15Δ0 can1::MFA1pr-HIS3 hht1-hhf1::NatMX4 hht2-hhf2::[HHTS-HHFS H4L97A]-URA3*	Open Biosystems	FY703
*MATa ura3Δ0 leu2Δ0 trp1Δ63 his3Δ200 lys2Δ0 met15Δ0 can1::MFA1pr-HIS3 hht1-hhf1::NatMX4 hht2-hhf2::[HHTS-HHFS H4Y98F]-URA3*	Open Biosystems	FY728
*BY4741 MATa his3Δ1 leu2Δ0 met15Δ0 ura3Δ0*		BY4741
*BY4741 MATa his3Δ1 leu2Δ0 met15Δ0 ura3Δ0 ilv1Δ::KanMX4*	Euroscarf	FY820
*BY4741 MATa his3Δ1 leu2Δ0 met15Δ0 ura3Δ0 aan1Δ::KanMX4*	Euroscarf	FY814
*BY4741 MATa his3Δ1 leu2Δ0 met15Δ0 ura3Δ0 bat1Δ::KanMX6*	This study	SY1143
*BY4741 MATa his3Δ1 leu2Δ0 met15Δ0 ura3Δ0 bat2Δ::KanMX4*	Euroscarf	FY815
*MATa ura3Δ0 leu2Δ0 trp1Δ63 his3Δ200 lys2Δ0 met15Δ0 can1::MFA1pr-HIS3 hht1-hhf1::NatMX4 hht2-hhf2::[HHTS-HHFS H3-WT]-URA3* [pRS424-BAT2]	This study	SY1164
*MATa ura3Δ0 leu2Δ0 trp1Δ63 his3Δ200 lys2Δ0 met15Δ0 can1::MFA1pr-HIS3 hht1-hhf1::NatMX4 hht2-hhf2::[HHTS-HHFS H3K4A]-URA3* [pRS424-BAT2]	This study	SY1166
*MATa ura3Δ0 leu2Δ0 trp1Δ63 his3Δ200 lys2Δ0 met15Δ0 can1::MFA1pr-HIS3 hht1-hhf1::NatMX4 hht2-hhf2::[HHTS-HHFS H3K4R]-URA3* [pRS424-BAT2]	This study	SY1168
*MATa ura3Δ0 leu2Δ0 trp1Δ63 his3Δ200 lys2Δ0 met15Δ0 can1::MFA1pr-HIS3 hht1-hhf1::NatMX4 hht2-hhf2::[HHTS-HHFS H3K121A]-URA3* [pRS424-BAT2]	This study	SY1172
*MATa ura3Δ0 leu2Δ0 trp1Δ63 his3Δ200 lys2Δ0 met15Δ0 can1::MFA1pr-HIS3 hht1-hhf1::NatMX4 hht2-hhf2::[HHTS-HHFS H3-WT]-URA3 bat1Δ::KanMX6*	This study	SY1216
*MATa ura3Δ0 leu2Δ0 trp1Δ63 his3Δ200 lys2Δ0 met15Δ0 can1::MFA1pr-HIS3 hht1-hhf1::NatMX4 hht2-hhf2::[HHTS-HHFS H3K4A]-URA3 bat1Δ::KanMX6*	This study	SY1217
*MATa ura3Δ0 leu2Δ0 trp1Δ63 his3Δ200 lys2Δ0 met15Δ0 can1::MFA1pr-HIS3 hht1-hhf1::NatMX4 hht2-hhf2::[HHTS-HHFS H3K4R]-URA3 bat1Δ::KanMX6*	This study	SY1218
*MATa ura3Δ0 leu2Δ0 trp1Δ63 his3Δ200 lys2Δ0 met15Δ0 can1::MFA1pr-HIS3 hht1-hhf1::NatMX4 hht2-hhf2::[HHTS-HHFS H3K121A]-URA3 bat1Δ::KanMX6*	This study	SY1219
*MATa ura3Δ0 leu2Δ0 trp1Δ63 his3Δ200 lys2Δ0 met15Δ0 can1::MFA1pr-HIS3 hht1-hhf1::NatMX4 hht2-hhf2::[HHTS-HHFS H3-WT]-URA3 bat1Δ::KanMX6* [pRS424-BAT2]	This study	SY1221
*MATa ura3Δ0 leu2Δ0 trp1Δ63 his3Δ200 lys2Δ0 met15Δ0 can1::MFA1pr-HIS3 hht1-hhf1::NatMX4 hht2-hhf2::[HHTS-HHFS H3K4A]-URA3 bat1Δ::KanMX6* [pRS424-BAT2]	This study	SY1222
*MATa ura3Δ0 leu2Δ0 trp1Δ63 his3Δ200 lys2Δ0 met15Δ0 can1::MFA1pr-HIS3 hht1-hhf1::NatMX4 hht2-hhf2::[HHTS-HHFS H3K4R]-URA3 bat1Δ::KanMX6* [pRS424-BAT2]	This study	SY1223
*MATa ura3Δ0 leu2Δ0 trp1Δ63 his3Δ200 lys2Δ0 met15Δ0 can1::MFA1pr-HIS3 hht1-hhf1::NatMX4 hht2-hhf2::[HHTS-HHFS H3K121A]-URA3 bat1Δ::KanMX6* [pRS424-BAT2]	This study	SY1224
*MATa ura3Δ0 leu2Δ0 trp1Δ63 his3Δ200 lys2Δ0 met15Δ0 can1::MFA1pr-HIS3 hht1-hhf1::NatMX4 hht2-hhf2::[HHTS-HHFS H3-WT]-URA3* [pRS424-BAT1-BAT2]	This study	SY1330
*MATa ura3Δ0 leu2Δ0 trp1Δ63 his3Δ200 lys2Δ0 met15Δ0 can1::MFA1pr-HIS3 hht1-hhf1::NatMX4 hht2-hhf2::[HHTS-HHFS H3K4A]-URA3* [pRS424-BAT1-BAT2]	This study	SY1331
*MATa ura3Δ0 leu2Δ0 trp1Δ63 his3Δ200 lys2Δ0 met15Δ0 can1::MFA1pr-HIS3 hht1-hhf1::NatMX4 hht2-hhf2::[HHTS-HHFS H3K4R]-URA3* [pRS424-BAT1-BAT2]	This study	SY1332
*MATa ura3Δ0 leu2Δ0 trp1Δ63 his3Δ200 lys2Δ0 met15Δ0 can1::MFA1pr-HIS3 hht1-hhf1::NatMX4 hht2-hhf2::[HHTS-HHFS H3K121A]-URA3* [pRS424-BAT1-BAT2]	This study	SY1333
*MATa ura3Δ0 leu2Δ0 trp1Δ63 his3Δ200 lys2Δ0 met15Δ0 can1::MFA1pr-HIS3 hht1-hhf1::NatMX4 hht2-hhf2::[HHTS-HHFS H3K121A]-URA3 agp1Δ::KanMX6*	This study	SY1316
*MATa ura3Δ0 leu2Δ0 trp1Δ63 his3Δ200 lys2Δ0 met15Δ0 can1::MFA1pr-HIS3 hht1-hhf1::NatMX4 hht2-hhf2::[HHTS-HHFS H3K121A]-URA3 bap2Δ::KanMX6*	This study	SY1320
*MATa ura3Δ0 leu2Δ0 trp1Δ63 his3Δ200 lys2Δ0 met15Δ0 can1::MFA1pr-HIS3 hht1-hhf1::NatMX4 hht2-hhf2::[HHTS-HHFS H3K121A]-URA3 agc1Δ::KanMX6*	This study	SY1280
*MATa ura3Δ0 leu2Δ0 trp1Δ63 his3Δ200 lys2Δ0 met15Δ0 can1::MFA1pr-HIS3 hht1-hhf1::NatMX4 hht2-hhf2::[HHTS-HHFS H3-WT]-URA3 tor1Δ::KanMX6*	This study	SY1188
*MATa ura3Δ0 leu2Δ0 trp1Δ63 his3Δ200 lys2Δ0 met15Δ0 can1::MFA1pr-HIS3 hht1-hhf1::NatMX4 hht2-hhf2::[HHTS-HHFS H3K4A]-URA3 tor1Δ::KanMX6*	This study	SY1189
*MATa ura3Δ0 leu2Δ0 trp1Δ63 his3Δ200 lys2Δ0 met15Δ0 can1::MFA1pr-HIS3 hht1-hhf1::NatMX4 hht2-hhf2::[HHTS-HHFS H3K4R]-URA3 tor1Δ::KanMX6*	This study	SY1190
*MATa ura3Δ0 leu2Δ0 trp1Δ63 his3Δ200 lys2Δ0 met15Δ0 can1::MFA1pr-HIS3 hht1-hhf1::NatMX4 hht2-hhf2::[HHTS-HHFS H3K121A]-URA3 tor1Δ::KanMX6*	This study	SY1191
*MATa ura3Δ0 leu2Δ0 trp1Δ63 his3Δ200 lys2Δ0 met15Δ0 can1::MFA1pr-HIS3 hht1-hhf1::NatMX4 hht2-hhf2::[HHTS-HHFS H3P16A]-URA3*	Open Biosystems	FY618
*MATa ura3Δ0 leu2Δ0 trp1Δ63 his3Δ200 lys2Δ0 met15Δ0 can1::MFA1pr-HIS3 hht1-hhf1::NatMX4 hht2-hhf2::[HHTS-HHFS H3P30V]-URA3*	Open Biosystems	FY661
*MATa ura3Δ0 leu2Δ0 trp1Δ63 his3Δ200 lys2Δ0 met15Δ0 can1::MFA1pr-HIS3 hht1-hhf1::NatMX4 hht2-hhf2::[HHTS-HHFS H3K36A]-URA3*	Open Biosystems	FY622
*MATa ura3Δ0 leu2Δ0 trp1Δ63 his3Δ200 lys2Δ0 met15Δ0 can1::MFA1pr-HIS3 hht1-hhf1::NatMX4 hht2-hhf2::[HHTS-HHFS H3P38A]-URA3*	Open Biosystems	FY623
*MATa ura3Δ0 leu2Δ0 trp1Δ63 his3Δ200 lys2Δ0 met15Δ0 can1::MFA1pr-HIS3 hht1-hhf1::NatMX4 hht2-hhf2::[HHTS-HHFS H3-WT]-URA3 agc1Δ::KanMX6*	This study	SY1277
*MATa ura3Δ0 leu2Δ0 trp1Δ63 his3Δ200 lys2Δ0 met15Δ0 can1::MFA1pr-HIS3 hht1-hhf1::NatMX4 hht2-hhf2::[HHTS-HHFS H3-WT]-URA3 bap2Δ::KanMX6*	This study	SY1317
*MATa ura3Δ0 leu2Δ0 trp1Δ63 his3Δ200 lys2Δ0 met15Δ0 can1::MFA1pr-HIS3 hht1-hhf1::NatMX4 hht2-hhf2::[HHTS-HHFS H3-WT]-URA3 agp1Δ::KanMX6*	This study	SY1313
*MATa ura3Δ0 leu2Δ0 trp1Δ63 his3Δ200 lys2Δ0 met15Δ0 can1::MFA1pr-HIS3 hht1-hhf1::NatMX4 hht2-hhf2::[HHTS-HHFS H3K4A]-URA3 agc1Δ::KanMX6*	This study	SY1278
*MATa ura3Δ0 leu2Δ0 trp1Δ63 his3Δ200 lys2Δ0 met15Δ0 can1::MFA1pr-HIS3 hht1-hhf1::NatMX4 hht2-hhf2::[HHTS-HHFS H3K4A]-URA3 bap2Δ::KanMX6*	This study	SY1318
*MATa ura3Δ0 leu2Δ0 trp1Δ63 his3Δ200 lys2Δ0 met15Δ0 can1::MFA1pr-HIS3 hht1-hhf1::NatMX4 hht2-hhf2::[HHTS-HHFS H3K4A]-URA3 agp1Δ::KanMX6*	This study	SY1314
*MATa ura3Δ0 leu2Δ0 trp1Δ63 his3Δ200 lys2Δ0 met15Δ0 can1::MFA1pr-HIS3 hht1-hhf1::NatMX4 hht2-hhf2::[HHTS-HHFS H3K4R]-URA3 agc1Δ::KanMX6*	This study	SY1279
*MATa ura3Δ0 leu2Δ0 trp1Δ63 his3Δ200 lys2Δ0 met15Δ0 can1::MFA1pr-HIS3 hht1-hhf1::NatMX4 hht2-hhf2::[HHTS-HHFS H3K4R]-URA3 bap2Δ::KanMX6*	This study	SY1319
*MATa ura3Δ0 leu2Δ0 trp1Δ63 his3Δ200 lys2Δ0 met15Δ0 can1::MFA1pr-HIS3 hht1-hhf1::NatMX4 hht2-hhf2::[HHTS-HHFS H3K4R]-URA3 agp1Δ::KanMX6*	This study	SY1315
*MATa ura3Δ0 leu2Δ0 trp1Δ63 his3Δ200 lys2Δ0 met15Δ0 can1::MFA1pr-HIS3 hht1-hhf1::NatMX4 hht2-hhf2::[HHTS-HHFS H3-WT]-URA3 bat1Δ::TRP1 bat2Δ::KanMX6*	This study	SY1308
*MATa ura3Δ0 leu2Δ0 trp1Δ63 his3Δ200 lys2Δ0 met15Δ0 can1::MFA1pr-HIS3 hht1-hhf1::NatMX4 hht2-hhf2::[HHTS-HHFS H3K4A]-URA3 bat1Δ::TRP1 bat2Δ::KanMX6*	This study	SY1309
*MATa ura3Δ0 leu2Δ0 trp1Δ63 his3Δ200 lys2Δ0 met15Δ0 can1::MFA1pr-HIS3 hht1-hhf1::NatMX4 hht2-hhf2::[HHTS-HHFS H3K4R]-URA3 bat1Δ::TRP1 bat2Δ::KanMX6*	This study	SY1310
*MATa ura3Δ0 leu2Δ0 trp1Δ63 his3Δ200 lys2Δ0 met15Δ0 can1::MFA1pr-HIS3 hht1-hhf1::NatMX4 hht2-hhf2::[HHTS-HHFS H3K121A]-URA3 bat1Δ::TRP1 bat2Δ::KanMX6*	This study	SY1311

**Oligonucleotides**		

TGTTAGCAAAATTACGGACGCC	This study	AAN1-RT-For (1315–1336)
CGGACCACCCTTTTCAGACA	This study	AAN1-RT-Rev (1395–1414)
GGTGGTGGTTTAATTGCTGG	This study	ILV1-RT-For (1715–1734)
GAGTTCTCTGGTTGCGTTGC	This study	ILV1-RT-Rev (1822–1841)
TCCAAGGTTGCCAACGACACAG	This study	ILV2-RT-For (2441–2462)
TGTTGAGCAGCCCACATTTGATG	This study	ILV2-RT-Rev (2498–2520)
TTGCACCTCCACCTCGTTACAC	This study	ILV3-RT-For (2667–2688)
ACCGTTGGAAGCGTTGGAAACC	This study	ILV3-RT-Rev (2719–2740)
TTACGCCGTCTGGAACGATGTC	This study	ILV5-RT-For (1648–1669)
GAACCAATGGCAACGGCCAAAG	This study	ILV5-RT-Rev (1698–1719)
TACCATGGTGCGTTGCAGTTCC	This study	ILV6-RT-For (1057–1078)
AGGTCTTGTTGCGTGTCTGTGC	This study	ILV6-RT-Rev (1111–1132)
TGAGTGATGCCAGACAAGGT	This study	LEU1-RT-For (1338–1357)
CCGTGAGTAGAGGTATGAGAGT	This study	LEU1-RT-Rev (1422–1443)
ACCATACGGCGGTGACTTG	This study	LEU4-RT-For (2062–2080)
TTCACCTTGAGCACGCTTCT	True et al.^51^	LEU4-RT-Rev (2145–2164)
AGAAATTGAACCCAGAGCGT	True et al.^51^	LEU9-RT-For (2460–2479)
TTCAGTAAATTGGATAGCGCA	True et al.^51^	LEU9-RT-Rev (2566–2586)
ACCCAAATCCATCCAAGCCA	Sing et al.^27^	BAT1-RT-For (1098–1117)
CACCCTTCTTTGGCTGACCA	Sing et al.^27^	BAT1-RT-Rev (1178–1197)
TTTCCACGCCTGATAGAGCC	Sing et al.^27^	BAT2-RT-For (1467–1486)
CAGTGGCTTCCAGTCTGACC	Sing et al.^27^	BAT2-RT-Rev (1543–1562)
ATCTTATTCCTATTCTTGGCTACC	Palavecino et al.^52^	AGP1-RT-For (2069–2092)
CGGCGTTAATGAAGTGTGG	^52^	AGP1-RT-Rev (2213–2231)
TAGAGGATGGCGTTGAGTC	Palavecino et al.^52^	BAP2-RT-For (1233–1251)
ACCAAGATGTAACCAATTATTAGC	Palavecino et al.^52^	BAP2-RT-Rev (1384–1407)
CATGATCAGATGGGGCTTGA	Nakanishi et al.^53^	PDC1-RT-For (2368–2387)
GAGCCTTTGGACCGTGAATC	Nakanishi et al.^53^	PDC1-RT-Rev (2437–2456)
GATCAGAAACGCCACCTTCC	Nakanishi et al.^53^	PDC5-RT-For (1945–1964)
CTGGGACAGCAACAGGTTTG	Nakanishi et al.^53^	PDC5-RT-Rev (2032–2051)
GCCTCACGCAGAGTACAACG	Nakanishi et al.^53^	PDC6-RT-For (2446–2465)
TGGCGATCTTGTGATTTTCG	Nakanishi et al.^53^	PDC6-RT-Rev (2521–2540)
GCAAGCTCTTTGGGGATCTG	Nakanishi et al.^53^	THI3-RT-For (2257–2276)
TTGCGGGGAATTTATCCTTG	^53^	THI3-RT-Rev (2332–2351)
AACGCTCACATCAATGGT	Wang et al.^54^	ARO10-RT-For (2468–2485)
ATGGTGCTCAGTTCTTGG	Wang et al.^54^	ARO10-RT-Rev (2557–2574)
AATGACTGATGGGGGTCTGG	Nakanishi et al.^53^	SFA1-RT-For (1792–1811)
GGCAGCCACACCAATGATAA	Nakanishi et al.^53^	SFA1-RT-Rev (1893–1912)
AGGCCACTGACGGTGGTGC	Vaudano et al.^55^	ADH1-RT-For (1701–1719)
AGCTGGCATACCGACCAAAACGG	Vaudano et al.^55^	ADH1-RT-Rev (1797–1819)
CTGCTGGTGGTCTAGGTTC	Wang et al.^54^	ADH2-RT-For (1536–1554)
CCGAGCGAGGTAAACAATTC	Wang et al.^54^	ADH2-RT-Rev (1625–1644)
AAGCCGCCAAAATTCAACAG	Nakanishi et al.^53^	ADH3-RT-For (1488–1507)
ACCAGAGATGGCAACCCAGT	Nakanishi et al.^53^	ADH3-RT-Rev (1596–1615)
GTTCAAGAGGCCAACATGCA	Mizuno et al.^56^	ADH4-RT-For (1865–1884)
TCTTGAGAAGCACCGAAATGC	Mizuno et al.^56^	ADH4-RT-Rev (1924–1944)
TCGATGGTGGTAATGCCAAG	Nakanishi et al.^53^	ADH5-RT-For (1611–1630)
ACTCCATGAGAACCGCCATT	Nakanishi et al.^53^	ADH5-RT-Rev (1718–1737)
GCCCATCCGATCCATATTGC	This study	BAT1-PRO-For (803–822)
AGGTTCTAGCTGATTTTGCGT	This study	BAT1-PRO-Rev (966–986)
TCCAACGAATCACCTCACCG	This study	ILV6-PRO-For (825–844)
TGTACTTCCATATATACCATTCCCC	This study	ILV6-PRO-Rev (920–944)
TTCCTTCCTTCATTCACGCACACT	Ahn et al.^57^	ADH1-PRO-For (765–788)
GTTGATTGTATGCTTGGTATAGCTTG	Ahn et al.^57^	ADH1-PRO-Rev (962–987)

**Recombinant DNA**		

2μm ori*,* Amp^R^*, TRP1*	Christianson et al.^58^	pRS424
2μm ori*,* Amp^R^*, TRP1, BAT2-HA*	This study	pRS424-BAT2
2μm ori*,* Amp^R^*, TRP1, BAT1-myc, BAT2-HA*	This study	pRS424-BAT1-BAT2

**Software and algorithms**		

ImageJ	Schneider et al.	
PyMOL Molecular Graphics System	Schrödinger, Inc	Version 3.1
Image Lab	Bio-Rad	Version 6
Prism	GraphPad Software, Inc.	Version 10

### Experimental model and study participant details

#### Yeast strains

The yeast strains used in this study are listed in STAR Methods. The standard lithium acetate method was used for yeast transformation. Disruption cassettes for gene deletion were constructed by PCR using genomic DNA of the corresponding strain obtained from Euroscarf. All strains were verified by PCR and/or immunoblot analysis. The H3 and H4 mutant strains used in this study were obtained from the Yeast Synthetic Histone H3 and H4 Mutant Collection (Open Biosystems), and each mutant strain was confirmed by DNA sequencing.

### Method details

#### Plasmids

The plasmids used in this study were constructed as previously described^59^ and are listed in STAR Methods. To generate pRS424-BAT2, the *BAT2* gene (with a C-terminal triple HA tag and 900 bp upstream and 700 bp downstream sequences) was PCR-amplified from SY1145 and cloned into pRS424. To create pRS424-BAT1-BAT2, the *BAT1* ORF, including 900 bp upstream and 800 bp downstream, was PCR-amplified from SY1276 and cloned into pRS424-BAT2.

#### Analysis of replicative lifespan

The replicative lifespan (RLS) of yeast strains was measured on rich glucose-based media (YPD) or SC plates unless otherwise indicated, as previously described.^59^ For SC plates, standard concentrations of branched-chain amino acids (BCAAs) were used: leucine (0.89 mM), isoleucine (0.80 mM), and valine (0.89 mM). Approximately 50 virgin daughter cells were subjected to RLS analysis unless otherwise noted. RLS screening was conducted using a multi-step approach. Initially, screening was performed with 5–6 mother cells per strain to identify candidates with potential lifespan differences. Strains whose mean RLS differed from wild-type by less than ±20% were classified as having no significant extension (NSE) or no significant decrease (NSD). Strains showing more considerable differences were further analyzed using 20 mother cells to confirm the initial findings. Those with a 20–50% reduction in mean RLS were classified as short-lived (SL), and those with more than a 50% reduction were classified as significantly short-lived (SSL). Strains that still showed <20% deviation from wild-type were again classified as NSE or NSD. For candidate long-lived (LL) strains, a final validation step was performed using 80 mother cells per strain. Strains with ≥20% increase in mean RLS compared to wild-type were classified as LL. Lifespan differences were assessed using the Mann–Whitney test, with a significance cutoff of *p* < 0.05. Comparison values between the control and each mutant strain are provided in STAR Methods.

#### Definition of nucleosome geographical domains

The assignment of the four geographical domains for each histone residue was determined as previously described^20^ with minor modifications. We obtained the accessible surface area (ASA) for each residue with DNA (ASA+) and without DNA (ASA-) and followed as described previously. After comparing ASA+ and ASA- of each amino acid residue, we classified residues as lateral residues when the difference was more significant than 9 Å. If the average ASA exceeded 10 Å, the residue was classified as 'disk'; otherwise, it was classified as 'buried'. Tail residues were manually assigned as H3 residues 1–37 and 135 and H4 residues 1–17. We used PyMOL (www.pymol.org/) to calculate all ASAs. To determine histone residue positions accurately, we used the yeast nucleosome crystal structure 4JJN [PBMID: 23650358], excluding the Sir3 protein.^60^ The crystal structure of the human nucleosome, 1KX5 [PBMID: 12079350], was aligned for comparative analysis. The dyad axis was defined as the specific DNA sequence where an association of the (H3/H4)_2_ tetramer with DNA nucleates nucleosome assembly.^61^

#### Measurement of intracellular branched-chain amino acids

BCAA levels were measured as previously described.^27^ Yeast cells were grown to mid-log phase (OD_600_ = 0.6–0.8) and harvested. Cells were washed three times with ice-cold PBS to remove residual media components. Cell lysates were prepared by vortexing the cells with glass beads in ice-cold PBS for 5 min. The cell debris and beads were then pelleted from the suspension at 13,000 rpm for 1 min at 4°C, and the supernatant was collected and placed on ice. Total protein concentration was determined using the Bradford assay. BCAA levels were measured using the Branched Chain Amino Acid Assay kit (Cell Biolabs Inc., San Diego, CA), following the manufacturer’s instructions. Measurements were performed in technical triplicates using negative control samples lacking leucine dehydrogenase. Free BCAAs were normalized to the total protein concentration from the cell lysates, and intracellular free BCAA concentrations were calculated based on leucine standards.

#### Growth assay

Growth rates were measured using a BioTek Synergy H1 Hybrid reader and a spectrophotometer. Yeast strains were grown to mid-log phase (OD_600_ = 0.5–0.6) in YPD at 30°C with constant shaking. Cells were then diluted to an initial OD_600_ of 0.1. For the BCAA metabolite experiment, growth was measured over 36 h in a modified SC medium containing only 10% of the standard BCAA concentrations (0.089 mM Leu, 0.080 mM Ile, 0.089 mM Val) and lacked glutamate. To assess the effects of BCAA metabolites, additional conditions were tested in which individual BCAAs (Leu, Ile, Val) or their corresponding keto forms (KIC, KIV, KMV) were supplemented to match the standard SC concentration (100%). In some conditions, glutamate or its keto form, α-ketoglutarate (α-KG), was also added to evaluate their combined effect on growth. All growth assays were performed in biological triplicates to ensure reproducibility and accuracy.

#### Spotting assay

The spotting assay was performed as previously described.^59^ Exponentially growing liquid cultures were normalized to an OD_600_ of 0.1 and subjected to 10-fold serial dilutions. 5 μL of each dilution was spotted onto YPD plates unless otherwise indicated, and the plates were incubated at 25°C, 30°C, or 37°C for 2 to 5 days. For sulfometuron methyl (SM) treatment, 8 μL of each dilution was spotted onto SC-Ile/Val plates containing 10 μM SM, and the plates were incubated at 30°C for 2 to 7 days. For growth defect restoration experiments, 0.80 mM of Ile and 0.89 mM of Val were added to the SC-Ile/Val media, representing 100% of the standard concentration. Additionally, Ile and Val were added at 100%, 200%, 500%, and 1000%. Experiments were also performed by supplementing the media with Ile or Val alone at 100%.

#### Rapamycin recovery assay

The rapamycin recovery assay was performed as previously described.^62^ Yeast cells were grown to mid-log phase (OD_600_ = 0.6–0.8) in YPD at 30°C with constant shaking. Upon reaching the desired OD_600_, 200 nM rapamycin was added to the culture, followed by a 2-h incubation. Following rapamycin treatment, cells were harvested and washed three times with distilled water to remove residual rapamycin. The prepared cells were then used for further analysis.

#### RNA isolation and reverse transcription-polymerase chain reactions (RT-PCRs)

Yeast cells were grown to an OD_600_ of 0.6–0.8 in 10 mL YPD at 30°C. Total RNA was extracted from the culture using the hot phenol method, as described previously.^9^ Reverse transcription was performed using 1 μg of total RNA and the DiaStar RT kit (Solgent). For quantitative PCR, cDNA was diluted 1:100 and used to quantify the expression of the target genes in Figure 4B (*AGP1, BAP1, ILV1, ILV2, ILV3, ILV5, ILV6, LEU1, LEU4, LEU9, BAT1, BAT2, AAN1*) and Figure 4C (*PDC1, PDC5, PDC6, THI3, ARO10, SFA1, ADH1, ADH2, ADH3, ADH4, ADH5*). Primer sequences used for qPCR are listed in STAR Methods. *ACT1* was used as the reference gene for normalization. Each qPCR reaction was performed in technical triplicates using the SsoAdvanced Universal SYBR Green Supermix (Bio-Rad). Relative RNA levels were calculated using the comparative Ct (ΔΔCt) method.^63^

#### Immunoblotting

Whole yeast cell extracts were prepared as previously described.^64^ Protein samples were separated by SDS-PAGE using either 10% or 15% polyacrylamide gels, depending on the target proteins. For detection of Rps6 phosphorylation, 10% gels were employed, while 15% gels were used for histone detection. Electrophoresis was conducted at 160V for 70 min. Proteins were transferred onto Immobilon-P PVDF membranes (Millipore) using a wet transfer system at 300 mA for 1 h 30 min on ice. Membranes were blocked with 5% BSA in TBS-T (20 mM Tris-HCl, 150 mM NaCl, 0.05% Tween 20, pH 7.6) for 1 h at room temperature. Subsequently, membranes were incubated overnight at 4°C with primary antibodies diluted in blocking buffer. After three washes with TBS-T, membranes were incubated with HRP-conjugated secondary antibodies diluted in blocking buffer for 1 h at room temperature. Following three additional washes, signals were detected using ECL detection reagent (GE Healthcare) and visualized with a ChemiDoc Imaging System (Bio-Rad) through chemiluminescence. Detailed information on the antibodies used is provided in the key resources table.

#### Re-analysis of RNA-seq and ATAC-seq data

For the analysis of histone mutant K121A, RNA-seq datasets were downloaded from the NCBI (SRA accession numbers: SRR12926683, SRR12926684, SRR12926685 and SRR12926686 for wild-type; SRR12926606, SRR12926607, and SRR12926608 for K121A).^19^ For the analysis of *set1Δ*, RNA-seq datasets were downloaded from an independent study (SRR2517451, SRR2517456 and SRR2517461 for *set1Δ*; SRR2517449, SRR2517454 and SRR2517459 for the corresponding wild-type controls).^20^ Raw SRA files were converted to FASTQ format using the SRA Toolkit. RNA-seq reads were aligned to the *Saccharomyces cerevisiae* reference genome (sacCer3, release R64-2-1) using HISAT2. The resulting SAM files were converted to BAM, sorted, and indexed using SAMtools. Ribosomal RNA (rRNA)-aligned reads were removed prior to quantification. Gene-level counts were generated using htseq-count. Differential expression analysis was conducted using DESeq2 in R. Volcano plots were generated using ggplot2 with custom thresholds for log_2_ fold change and adjusted *p*-value.

For the re-analysis of ATAC-seq data, ATAC-seq datasets were downloaded from the NCBI (SRA file accession numbers SRR13963722 and SRR13963723).^21^ ATAC-seq reads were aligned to the *Saccharomyces cerevisiae* reference genome (sacCer3, release R64-2-1) using Bowtie2. Aligned reads were indexed and filtered using Sambamba to remove reads aligned to chrM, duplicate reads, improperly paired reads, and those with low mapping quality (<30). HMMRATAC was used for peak calling with the "--trim 1" parameter” to remove the 3 N signal track. Heatmaps were generated using deepTools.

#### Chromatin immunoprecipitations

Chromatin immunoprecipitations were performed as previously described.^22^^,^^23^ Yeast strains were grown to an OD_600_ of approximately 0.6 and crosslinked for 20 min by adding formaldehyde to a final concentration of 1%. Crosslinking was quenched by adding glycine to a final concentration of 240 mM. Antibodies directed against H3K4me3 (Abcam, ab8580) or H3 (Abcam, ab1791) were bound to protein A-Sepharose CL-4B (GE Healthcare, GE17-0780-01) to precipitate chromatin. Diluted template DNA (1:2 and 1:500 dilutions for IP and input DNA, respectively) was used in qPCR reactions with the SsoAdvanced Universal SYBR Green Supermix (Bio-Rad). All signals for H3K4me3 were further normalized to the input DNA and their respective total histone H3 signals. Primer sequences used for qPCR are listed in STAR Methods.

### Quantification and statistical analysis

Statistical analyses were performed using GraphPad Prism 8 software. Data are presented as mean ± standard error of the mean (SEM), unless otherwise specified. Statistical *p*-values for two-group comparison were conducted by a two-tailed Student’s t test (parametric) and a Mann-Whitney test (non-parametric) results. For all statistical tests, *p*-values are denoted as followed: ∗*p* < 0.05; ∗∗*p* < 0.01; ∗∗∗*p* < 0.001. Additional details regarding statistical analyses are provided in the figure legends.

## Supplemental information

Document S1. Figures S1–S9 and Data S1

Table S1. Mean lifespans and *p* values, related to Figures 1–7 and S5–S9
