# Supplementary material for: Epigenetic regulation of intracellular branched-chain amino acid homeostasis maintains a normal lifespan
Source: iScience. 2025 Jun 7;28(7):112846. doi: 10.1016/j.isci.2025.112846 (PMC12246581; doi:10.1016/j.isci.2025.112846)
Supplement: Document S1. Figures S1–S9 and Data S1 [file mmc1.pdf]

iScience, Volume *28*

## Supplemental information

# Epigenetic regulation of intracellular branched-chain amino acid homeostasis maintains a normal lifespan

**Sejung Park, Yan Liu, Suji Lim, Hong-Yeoul Ryu, and Seong Hoon Ahn**

Park/Liu *et al.*, Supplementary Figure S1

**A**

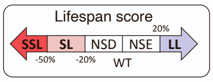


| RLS Relative to WT, % | Classification | H3 mutants (% relative to the 187 mutants) | H4 mutants (% relative to the 138 mutants) |
|---|---|---|---|
| ≥20% | LL (Long-Lived) | 2 (1%) | 0 (0%) |
| ≥0%, <20% | NSE (Not-Significantly-Extended) | 27 (14%) | 24 (17%) |
| <0%, >-20% | NSD (Not-Significantly-Decreased) | 84 (45%) | 52 (38%) |
| ≤-20%, >-50% | SL (Short-Lived) | 42 (23%) | 51 (37%) |
| ≤-50% | SSL (Significantly-Short-Lived) | 32 (17%) | 11 (8%) |
| Total LSL strains | | 76 (41%) | 62 (45%) |

**B**

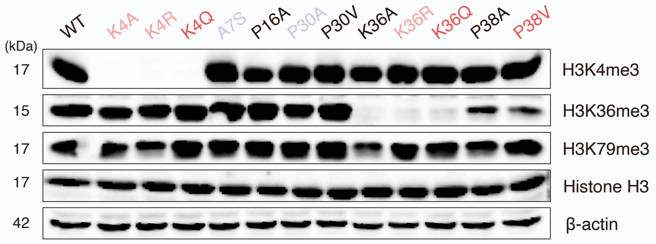

Park/Liu *et al.*, Supplementary Figure S2

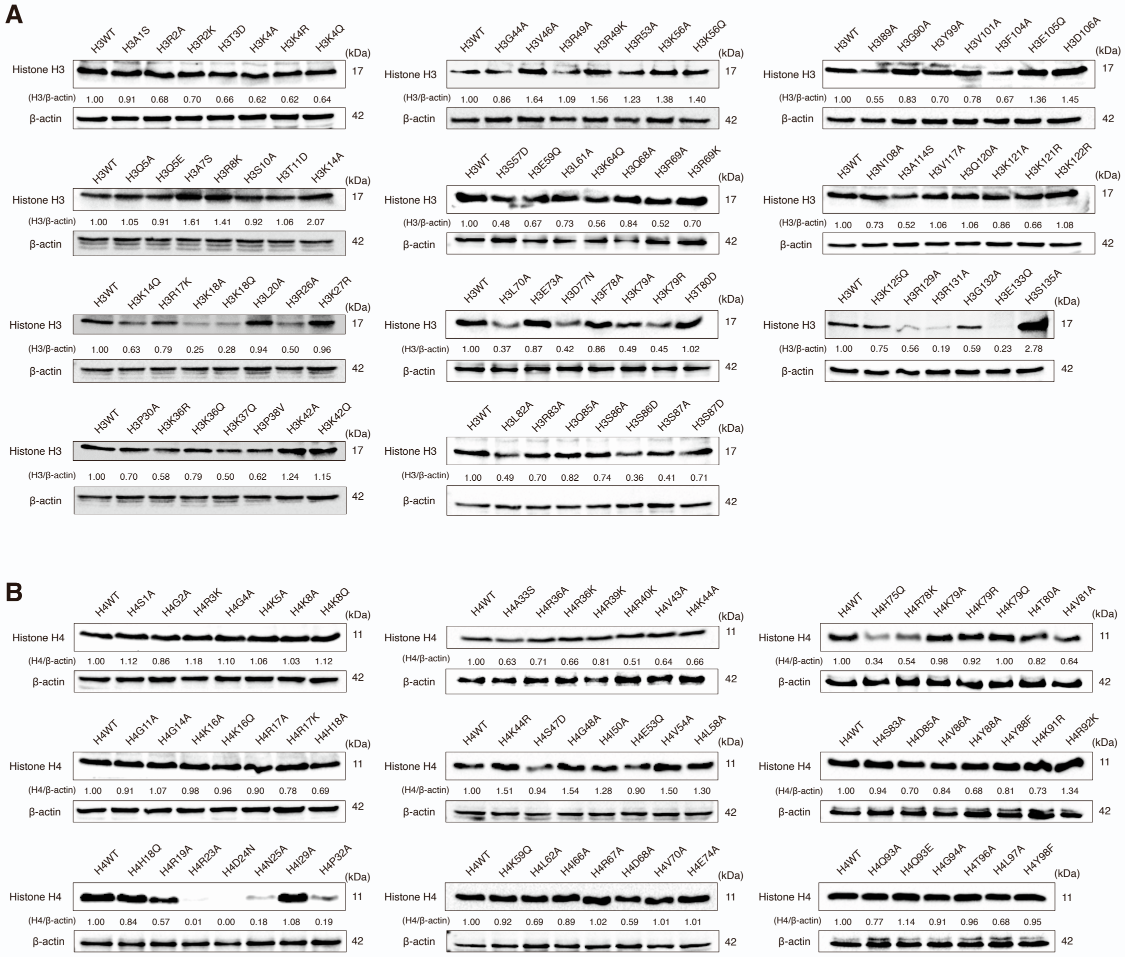

Park/Liu *et al.*, Supplementary Figure S3

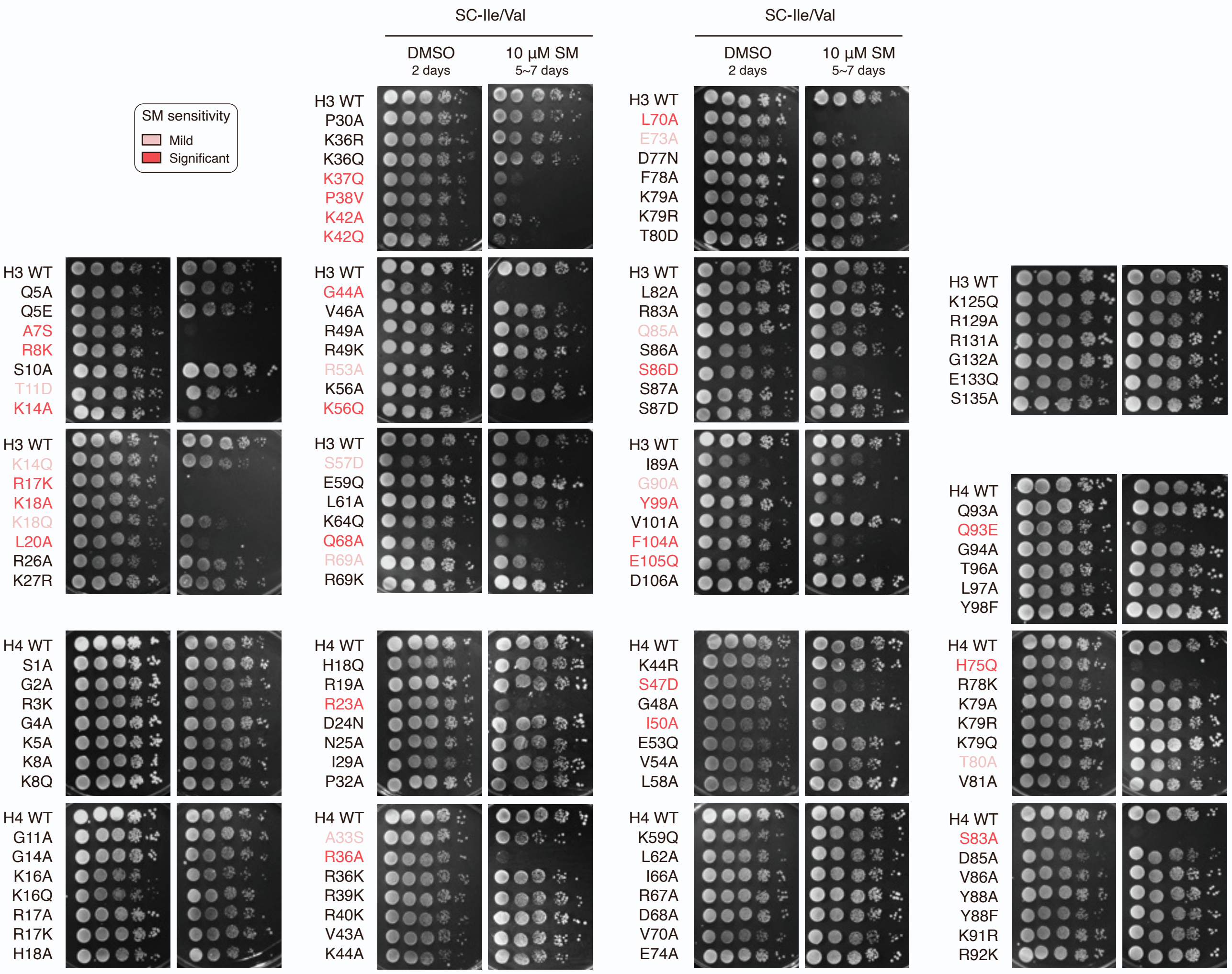

Park/Liu *et al.*, Supplementary Figure S4

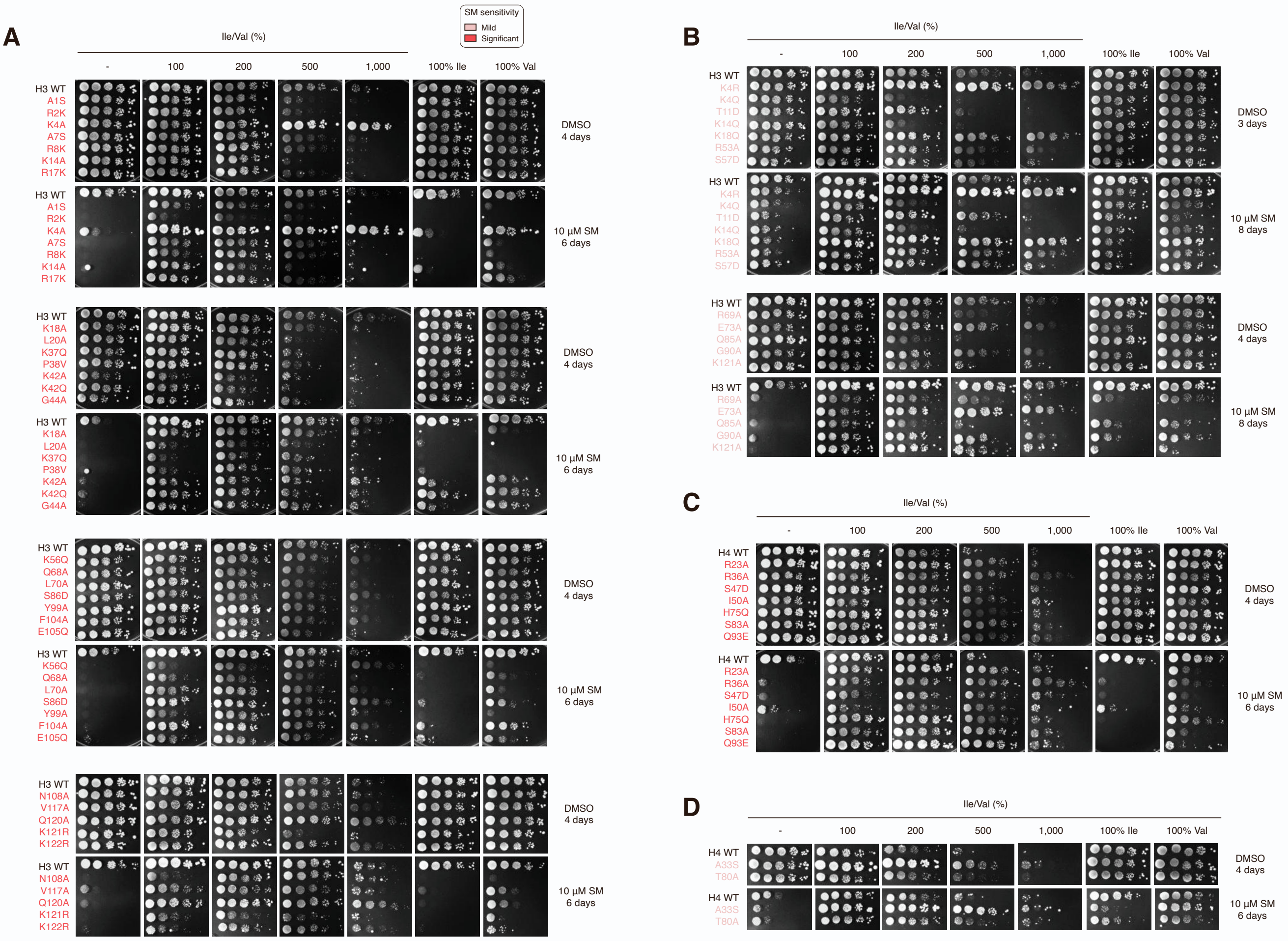

Park/Liu *et al.*, Supplementary Figure S5

**A**

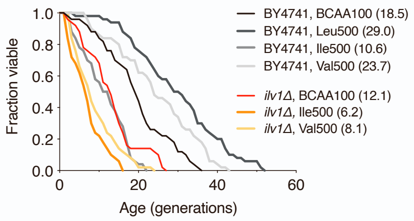


**B**

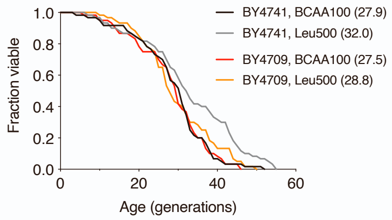

Park/Liu *et al.*, Supplementary Figure S6

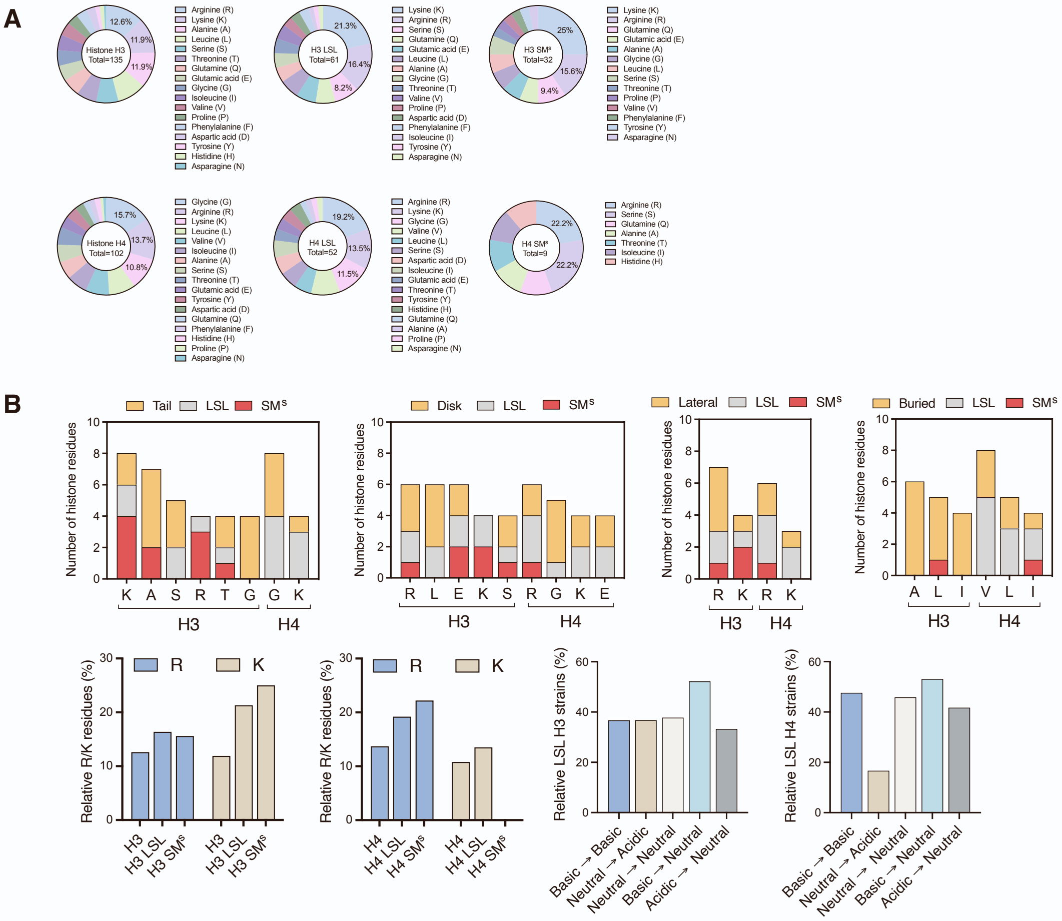

Park/Liu *et al.*, Supplementary Figure S7

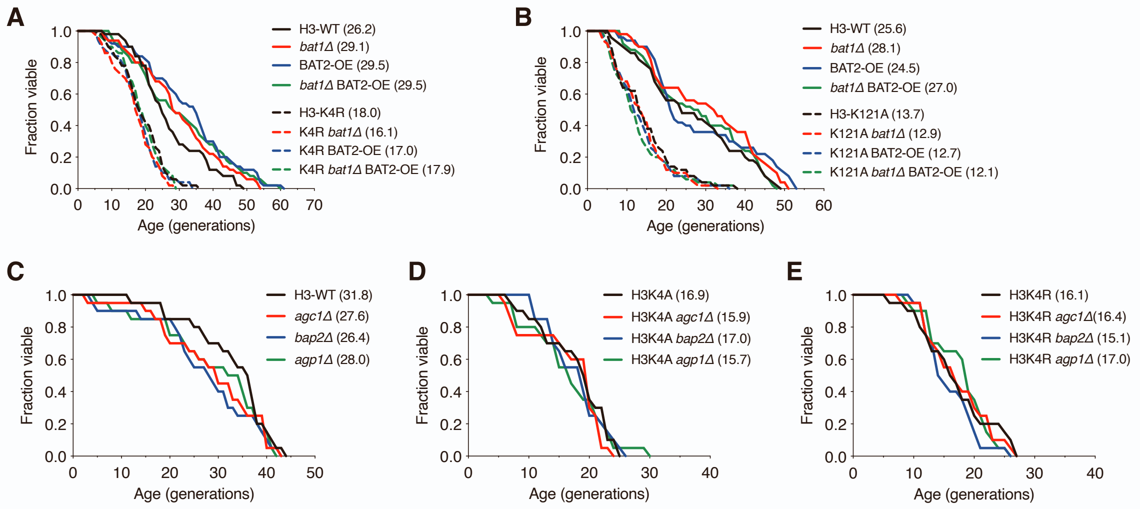

Park/Liu *et al.*, Supplementary Figure S8

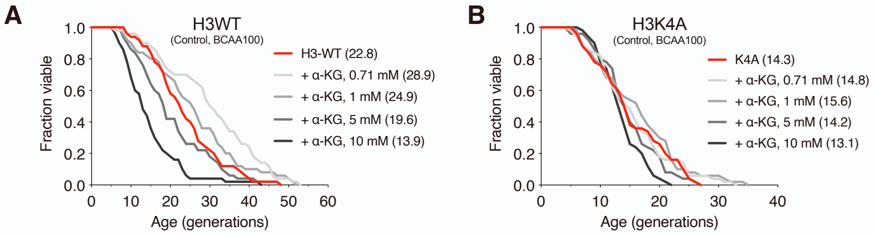

Park/Liu *et al.*, Supplementary Figure S9

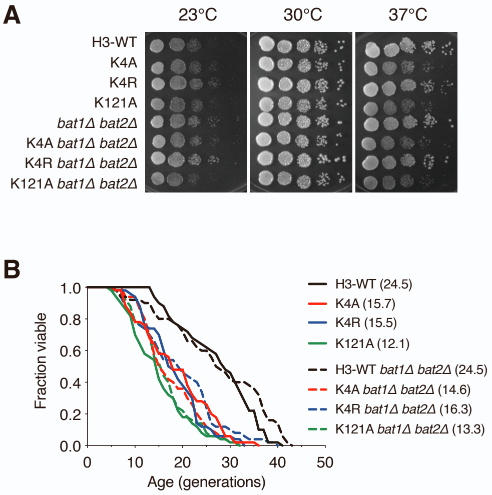

Data S1. Unprocessed Western blot images corresponding to Figures 6E, 6F, and 6G.

## Figure 6E

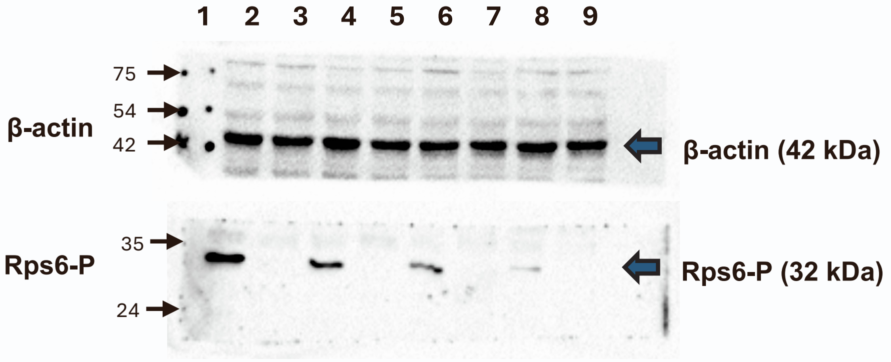


## Figure 6F

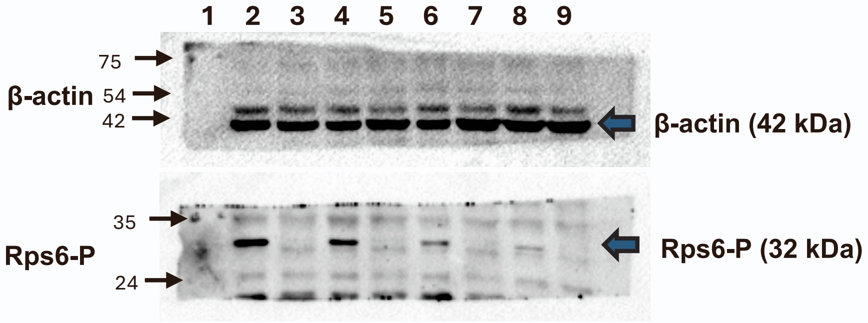


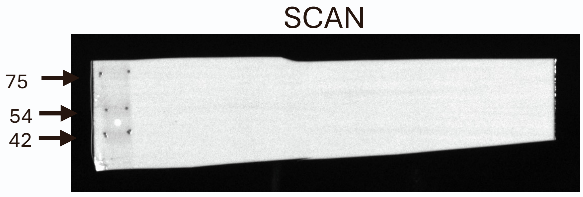


## Figure 6G

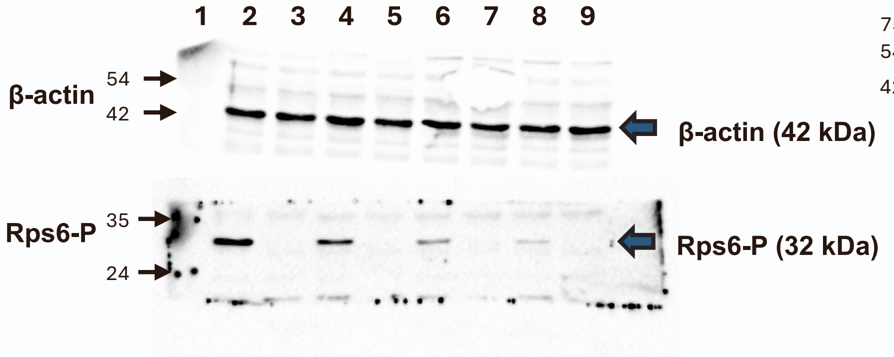


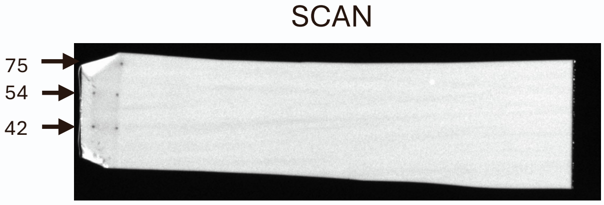

## SUPPLEMENTARY FIGURE LEGENDS

**Figure S1. Classification of H3/H4 mutants from the RLS screen and methylation analysis of histone H3 mutants including H3A7S and H3P30A, related to Figure 1**
(A) The RLS screening results in mutant cells from the yeast synthetic histone H3 and H4 mutant collection (Open Biosystems) are shown in the table, with the lifespan score used in Figure. 1C. The percentage relative to the total histone H3 or H4 mutants is shown in parenthesis.
(B) Levels of histone H3 trimethylation at Lys 4, Lys 36, or Lys79 were analyzed by Western blot. Results were normalized by both total histone and actin. The color codes of the LSL residues are in Figure. S1A.

**Figure S2. Western blot analysis using the histone H3 (A) or the H4 antibody (B), related to Figure 1E**

**Figure S3. Growth assays to measure SM-sensitivities of LSL strains, related to Figure 2B**

**Figure S4. Growth assays are used to determine the restoration of SM-induced growth defects by supplementing various concentrations of BCAAs, related to Figure 2A**
(A) Results of growth assay in the significantly sensitive H3 (A), the mildly sensitive H3 (B), significantly sensitive H4 (C), and the mildly sensitive H4 (D) strains, respectively.

**Figure S5. RLS analysis in the indicated strains, related to Figure 3**

**Figure S6. Residual analysis of the LSL strains, related to Figure 3**
(A) Pie charts showing the amino acid composition of histone H3 and H4 residues across different mutant categories; the residues in WT histone H3 or H4, those in LSL, or those in SM-sensitive LSL strains are shown.
(B) Bar graphs comparing the relative abundance of arginine (R) and lysine (K) residues in histone H3 and H4. Details are shown in the text.

**Figure S7. RLS analysis in the indicated strains, related to Figure 5**
For the experiments in Figures. S7C-S7E, each of the twenty virgin daughter cells was subjected to lifespan analyses. See text for details.

**Figure S8. RLS analysis in the indicated strains, related to Figure 7**

**Figure S9. BAT1 and BAT2 double deletion does not rescue the growth and replicative lifespan defects caused by H3K4A, H3K4R, or H3K121A, related to Figure 5**
(A) Spotting assays in the indicated strain at 23°C, 30°C, or 37°C.
(B) RLS analysis in the indicated strains. See text for details.

## DATA S1. FIGURE LEGENDS

**Figure 6E. The raw image of the Western blot analysis is in Figure 6E. Rps6 phosphorylation was determined after 2 h incubation with rapamycin or DMSO**

Lane 1, Pre-stained marker (ELPIS, Cat# EBM2000)
Lane 2, WT for histone H3
Lane 3, WT strain treated with 200 nM of rapamycin
Lane 4, The *tor1Δ* strain
Lane 5, The *tor1Δ* strain treated with 200 nM of rapamycin
Lane 6, The K4A strain
Lane 7, The K4A strain treated with 200 nM of rapamycin
Lane 8, The K4A *tor1Δ* strain
Lane 9, The K4A *tor1Δ* strain treated with 200 nM of rapamycin

**Figure 6F. The raw image of the Western blot analysis is in Figure 6F. Rps6 phosphorylation was determined after 2 h incubation with rapamycin or DMSO**

The scanned image of the β-actin blot in Figure 6F is shown on the right.
Lane 1, Pre-stained marker (ELPIS, Cat# EBM2000)
Lane 2, WT for histone H3
Lane 3, WT strain treated with 200 nM of rapamycin
Lane 4, The *tor1Δ* strain
Lane 5, The *tor1Δ* strain treated with 200 nM of rapamycin
Lane 6, The K4R strain
Lane 7, The K4R strain treated with 200 nM of rapamycin
Lane 8, The K4R *tor1Δ* strain
Lane 9, The K4R *tor1Δ* strain treated with 200 nM of rapamycin

**Figure 6G. The raw image of the Western blot analysis is in Figure 6G. Rps6 phosphorylation was determined after 2 h incubation with rapamycin or DMSO**

The scanned image of the β-actin blot in Figure 6G is shown on the right.
Lane 1, Pre-stained marker (ELPIS, Cat# EBM2000)
Lane 2, WT for histone H3
Lane 3, WT strain treated with 200 nM of rapamycin
Lane 4, The *tor1Δ* strain
Lane 5, The *tor1Δ* strain treated with 200 nM of rapamycin
Lane 6, The K121A strain
Lane 7, The K121A strain treated with 200 nM of rapamycin
Lane 8, The K121A *tor1Δ* strain
Lane 9, The K121A *tor1Δ* strain treated with 200 nM of rapamycin
